# Engineering astaxanthin accumulation reduces photoinhibition and increases biomass productivity under high light in *Chlamydomonas reinhardtii*

**DOI:** 10.1186/s13068-022-02173-3

**Published:** 2022-07-11

**Authors:** Stefano Cazzaniga, Federico Perozeni, Thomas Baier, Matteo Ballottari

**Affiliations:** 1 Department of Biotechnology, University of Verona, Strada le Grazie 15, 37134 Verona, Italy; 2 Faculty of Biology, Center for Biotechnology (CeBiTec), Bielefeld University, Universitätsstrasse 27, 33615 Bielefeld, Germany

**Keywords:** Microalgae, Astaxanthin, High light stress, Metabolic engineering, Carotenoids, Photosynthesis

## Abstract

**Background:**

Astaxanthin is a highly valuable ketocarotenoid with strong antioxidative activity and is natively accumulated upon environmental stress exposure in selected microorganisms. Green microalgae are photosynthetic, unicellular organisms cultivated in artificial systems to produce biomass and industrially relevant bioproducts. While light is required for photosynthesis, fueling carbon fixation processes, application of high irradiance causes photoinhibition and limits biomass productivity.

**Results:**

Here, we demonstrate that engineered astaxanthin accumulation in the green alga *Chlamydomonas reinhardtii* conferred high light tolerance, reduced photoinhibition and improved biomass productivity at high irradiances, likely due to strong antioxidant properties of constitutively accumulating astaxanthin. In competitive co-cultivation experiments, astaxanthin-rich *Chlamydomonas reinhardtii* outcompeted its corresponding parental background strain and even the fast-growing green alga *Chlorella vulgaris*.

**Conclusions:**

Metabolic engineering inducing astaxanthin and ketocarotenoids accumulation caused improved high light tolerance and increased biomass productivity in the model species for microalgae *Chlamydomonas reinhardtii*. Thus, engineering microalgal pigment composition represents a powerful strategy to improve biomass productivities in customized photobioreactors setups. Moreover, engineered astaxanthin accumulation in selected strains could be proposed as a novel strategy to outperform growth of other competing microalgal strains.

**Supplementary Information:**

The online version contains supplementary material available at 10.1186/s13068-022-02173-3.

## Background

Eukaryotic microalgae are diverse single-celled organisms capable to perform photosynthesis [1]. They can be found in all biological ecosystems and are typically grown in controlled bioreactors to use light for CO_2_ fixation into organic biomass and valuable bioproducts [2]. Despite their potential, industrial application of microalgae is limited by physiological and technical constrains that reduce the economic feasibility [3, 4]. Improving microalgae cultivation is a multivariable challenge that includes strain selection and optimization of cultivation process design, which can partially be addressed by improved light energy utilization for biomass and biomolecules production.

Oxygenic photosynthesis is carried out by four multi-subunit membrane–protein complexes in the thylakoid membrane: Photosystem I and II (PSI and PSII), cytochrome b6f and ATPase [5]. Each photosystem is composed by a core complex connected to an array of antenna complexes that increase their light harvesting properties [6]. Both antenna and core complexes include several protein subunits that binds pigments as chlorophylls (Chl) a, b and carotenoids. Light absorption by both photosystems fuels the electron transport from O_2_ to NADPH. This electron transport is mediated by several electron carriers coupling electron transfer with proton pumping from the stroma to the lumen, generating a transmembrane ΔpH gradient used by the ATP synthase enzyme to produce ATP. When light intensity is too high and electron transport is saturated, the photochemical quenching of Chl excited states is impaired, increasing the probability to generate Reactive Oxygen Species (ROS). Because of its strong oxidizing potentials, ROS induce damage in their local environment by destroying lipids, nucleic acids and proteins yielding into widespread oxidation and reducing biomass accumulation [7]. Microalgae have different protection mechanisms for excess light energy such as regulation of cellular pigments contents, reorganization of photosystems, redistribution of antenna proteins between PSI and PSII (state transitions) or accumulation of antioxidant molecules [8]. Short term photoprotective mechanisms such as thermal dissipation of the excitation energy absorbed in excess in a complex process called non-photochemical quenching (NPQ) [9, 10] can further assist in fluctuating light conditions. NPQ is activated by ΔpH on the thylakoid membrane and work as feedback-regulatory mechanism for excitation energy transfer to reaction centres. In *C. reinhardtii*, two LHC proteins, LHCSR1 and LHCSR3, were reported to have a key role in NPQ induction [11–14]. The accumulation of both isoforms is depended on the light regime with LHCSR1 present also in low light while LHCSR3 mainly accumulated in high light [15].

These native protection mechanisms are efficient in avoiding photodamage and increase fitness in natural ecosystems but are not tailored to achieve maximal biomass accumulation [16]. However, microalgae cultivation in artificial bioreactors is typically different from natural conditions. In industrially relevant cultivation systems, high cell densities are required often exposed to strong continuous illumination to maximize biomass production. Moreover, algal growth is inhibited by constantly changing irradiances due to the steep gradient in light intensity applied from outside of the bioreactor, where cells rapidly move between layers of low and high light, which increases the risk of cellular photodamage. Strategies directed at enhancing the resistance of microalgae to oxidative stress could likely increase their productivity [17].

Carotenoids, present in all photosynthetic organisms, where they play crucial roles in photosynthesis and photoprotection [18]. They are involved in photosystem assembly and light harvesting, contribute to the antioxidant network of the chloroplast, and detoxify ROS generated by photosynthesis preventing lipid peroxidation [19]. In the case of *Chlamydomonas reinhardtii* carotenes as β-carotene and α-carotene and xanthophylls as lutein, loroxanthin, zeaxanthin, violaxanthin and neoxanthin are involved in the photosynthetic process [20]. The stoichiometry of different carotenoids and their specific binding site to photosynthetic complex complexes are conserved in a wide range of taxa from microalgae to crops plants [18]. Despite this conserved distribution, in other species of algae different types of carotenoids can found. Diatoms and multicellular brown algae accumulate xanthophylls fucoxanthin, diadinoxanthin and diatoxanthin while some species of microalgae accumulate ketocarotenoids, like astaxanthin. Ketocarotenoids show a higher antioxidant capacity with respect to the carotenes and xanthophylls normally accumulating in green microalgae [21]. They are obtained by β-carotene ketolase (BKT, named also crtW) activity that catalyses the addition of a keto-group at the C4 position of carotenoids β-rings [22]. The most studied ketocarotenoids is astaxanthin that is synthetized adding a keto-group on both rings of zeaxanthin. Due to this carbonyl group this pigment has an antioxidant activity 10 times stronger than zeaxanthin and β-carotene [23, 24]. Higher plants and most microalgae do not possess the carotene ketolase activity and consequently do not synthesize ketocarotenoids. The most prominent source of astaxanthin is fresh-water microalgae *Haematococcus lacustris* which can accumulate astaxanthin up to 5% of its dry weight during hematocyst formation upon exposure to stress conditions such as excessive light, nutrient starvation, high salinity or high/low temperature [25]. *H. lacustris* cysts exhibited higher photoprotection capacity and resistance to pigment photobleaching [26].

Although the genome of *C. reinhardtii* harbours the genetic information for BKT [20], its expression is limited to diploid zygospore stage [27]. Recently, synthetic redesign of this gene enabled its constitutive overexpression and robust accumulation of ketocarotenoids in *C. reinhardtii* [28]. In this work, we investigated the effect of astaxanthin accumulation in *C. reinhardtii* and characterized resistance to oxidative stress and photoinhibition. Engineered *bkt* strains, were able to accumulate ketocarotenoids as their most abundant carotenoids in their thylakoids, demonstrated a higher resistance to photodamage and an improved performance in high light with respect to wild type. Remarkably, growth of *C. reinhardtii bkt* outcompeted the fast-growing green algae *Chlorella vulgaris* in high light conditions. These results demonstrate that inducing astaxanthin accumulation in microalgae could be a successful strategy to improve biomass accumulation in advanced microalgae cultivations.

## Results

Generation of *C. reinhardtii* strains overexpressing BKT enzyme, herein named *bkt* strains, was described previously [28]. In summary, *Cr*BKT was redesigned in silico, optimizing the amino acids (aa) codon usage, spreading of the RuBisCO small subunit II (RBCS2) intron 1 sequence to minimize exon lengths and increase transgene expression [29, 30] and removal of a redundant 116 aa-long C-terminal sequence extension [31]. *C. reinhardtii* strain UVM4 was previously engineered to enable robust protein production [32] and was used for *Cr*BKT expression. Among the transformant lines generated, *bkt5* strain was selected for the experiments herein described, being one of the strains with the higher rate of ketocarotenoids production [28]. In all experiments presented in this study, *bkt5* was used as ketocarotenoid-accumulating strain while UVM4 served as a parental control.

### Pigments accumulation and composition of photosynthetic apparatus

To study the physiologic effect of ketocarotenoids accumulation in *C. reinhardtii*, UVM4 and *bkt5* strains were grown photoautotrophically in high-salt (HS) medium [33] at two different light intensities: control (CL, 100 μmol photons/m^2^/s) and high light (HL, 600 μmol photons/m^2^/s). Pigments were extracted and analysed by spectral absorbance and HPLC (Additional file 1: Table S1). *bkt5* strain accumulated a high amount of ketocarotenoids, accounting for more than 50% of total carotenoids in CL and 59% in HL. In particular, a production of 0.26 ± 0.03 and 0.37 ± 0.06 mg/L of astaxanthin at the end of the growth, respectively, for cells grown in CL and HL were achieved, in line with previous finding [28]. The change in carotenoids composition affected the Chl content in the *bkt5*, with a 30% decrease in Chl content per cell in CL. In HL, both UVM4 and *bkt5* strains reduced their Chl content per cell, but the differences in terms of Chl content were stronger compared to the CL case, with a ~ 50% decrease in *bkt5* compared to UVM4. Total carotenoids content was also reduced in *bkt5* compared to the UVM4 case in CL and HL, respectively, with a 20% and 30% decrease. Chl a/Chl b and Chl/Car ratios were also significantly reduced in *bkt5* compared to UVM4 in both CL and HL conditions: since Chl b is bound only by antenna subunits reduced Chl a/b ratio indicate that *bkt5* had a different reorganization of the photosynthetic apparatus compared to the UVM4. Chl/Car ratios are reduced in HL compared to CL in both strains, in line with the higher carotenogenesis at strong irradiance [34], and further reduced in *bkt5*. HPLC analysis confirmed that ketocarotenoids were present in *bkt5* but not in the UVM4 (Additional file 1: Table S2). Astaxanthin biosynthesis starts from β carotene or zeaxanthin generating a series of different intermediate ketocarotenoids [22]. In *bkt5* astaxanthin was the main carotenoid accumulated but other ketocarotenoids intermediates as canthaxanthin, adonirubin, adonixanthin and hydroxyechinenone were also detected at lower contents. In HL *bkt5* was characterized by a general increase of ketocarotenoid content, with the strongest increase observed in the case of canthaxanthin (Additional file 1: Table S2). Other than ketocarotenoids, in *bkt5* there was a relative decrease of all the carotenoids normally present in *C. reinhardtii* with the strongest effect observed in the case of β-β xanthophylls as neoxanthin and violaxanthin.

**Fig. 1:**
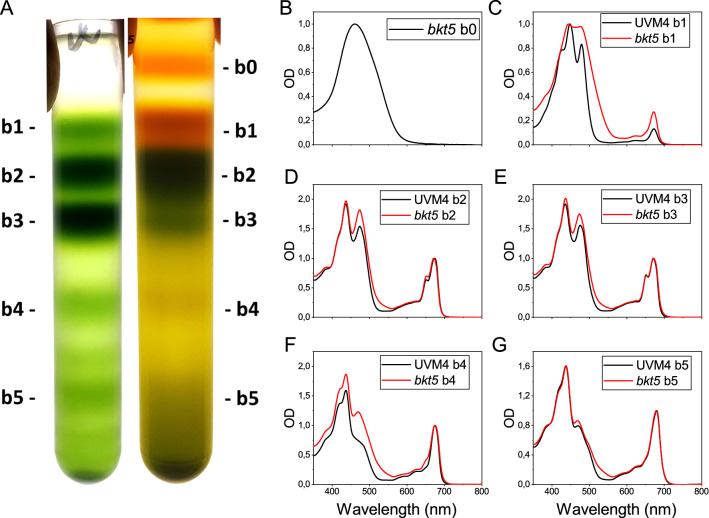
Distribution of pigment-binding complexes. **A** Ultracentrifugation in sucrose gradients of UVM4 and *bkt5* solubilized thylakoids isolated from cells adapted in HL. **B**–**G** Native absorption spectra of the different fraction; b0 and b1 were normalized to the maximal absorption, b2–b5 were normalized to absorption peak in the 600–740 nm region

The change in pigment composition in *bkt5* compared to UVM4 suggests a possible a reorganization of photosynthetic complexes. Composition of the photosynthetic apparatus was analysed by solubilization of thylakoid membranes with dodecyl‐α‐d‐maltoside and ultracentrifugation in sucrose gradients (Fig. 1). In UVM4, different green bands were recovered and identified by absorption spectra; from top to bottom, the bands corresponded to free pigments (b1), LHC protein in monomeric state (b2), trimeric LHCII (b3), PSII core complex (b4) and PSI‐LHCI (b5) [35]. In *bkt5* strain b1 band was highly enriched in carotenoids and there was an additional upper band (b0) composed of ~ 90% of ketocarotenoids (Additional file 1: Figure S1). Monomeric LHC fraction (b2) was more abundant than trimeric LHC (b3) in *bkt5*, while the opposite was observed in the case of UVM4, suggesting a destabilization of LHCII trimers upon accumulation of ketocarotenoids. PSII core and PSI fractions (b4 and b5) were reduced in *bkt5* compared to UVM4, in line with Chl a/Chl b reduction observed upon ketocarotenoids accumulation (Additional file 1: Table S1). The absorption spectra of the different fractions isolated from *bkt5* strain were characterized by a shoulder around 530 nm which is related to astaxanthin and ketocarotenoids absorption (Fig. 1).

**Fig. 2:**
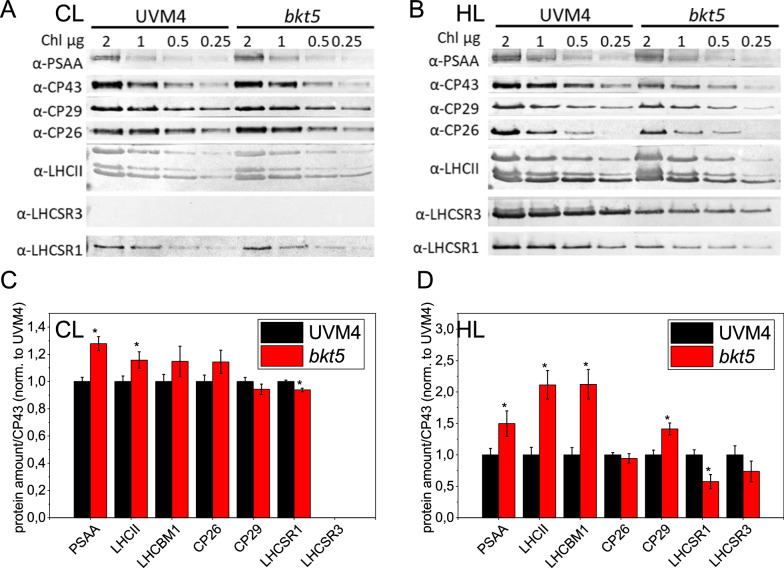
Immunoblotting analysis of photosynthetic subunits. Immunoblotting analysis of thylakoid proteins isolated from cells of UVM4 or *bkt5* cells, acclimated to control light (**A**) or high light (**B**). Specific antibodies against PSAA, LHCII, CP26, CP29, LHCSR1 and LHCSR3 were used on lanes loaded with 2, 1, 0.5 and 0.25 µg of Chls. **C**, **D** Densitometric analysis of immunoblotting results. Data are reported normalized to CP43 amount and set to 1 in the case of UVM4. Error bars are reported as standard deviation (*n* = 4). Values significantly different comparing *bkt5* with UVM4 are reported with * (Student’s *t* test, *P* < 0.05)

The HPLC analysis of the fractions of the *bkt5* gradient allowed studying ketocarotenoids distribution (Additional file 1: Figure S1). Half of the ketocarotenoids were accumulated mainly as free pigments in fractions b0 and b1, the remaining part was distributed in the gradient, especially in the fractions where antenna proteins migrated. The distribution of ketocarotenoids throughout the whole gradient might be related to binding of these carotenoids to different protein subunits, or to a contamination due to high astaxanthin accumulation in the thylakoid membrane. Photosynthetic apparatus of *bkt5* was then compared to the UVM4 case by immunoblotting of thylakoid membrane isolated from cell adapted in CL and HL by using specific antibodies recognizing PSI, PSII and LHC subunits (Fig. 2, Additional File 1 Figure S2). In CL, *bkt5* showed a ~ 30% increase in PSI/PSII ratio and  ~ 15% increase in LHCII/PSII compared to UVM4, while the stoichiometry of monomeric CP26 and CP29 per PSII were not significantly affected. These changes are in line with the lower Chl a/b ratio observed in *bkt5* compared to UVM4 being Chl b bound only by LHC antenna protein and having the PSI a lower chl b content with respect to PSII. The differences between *bkt5* and UVM4 were more severe in HL with a relative increase of PSI/PSII and LHCII/PSII, respectively, of ~ 50% and  ~ 120%, respectively. In HL a 40% increase of CP29/PSII ratio was also observed in the case of *bkt5*.

### Photosynthetic efficiency

**Fig. 3:**
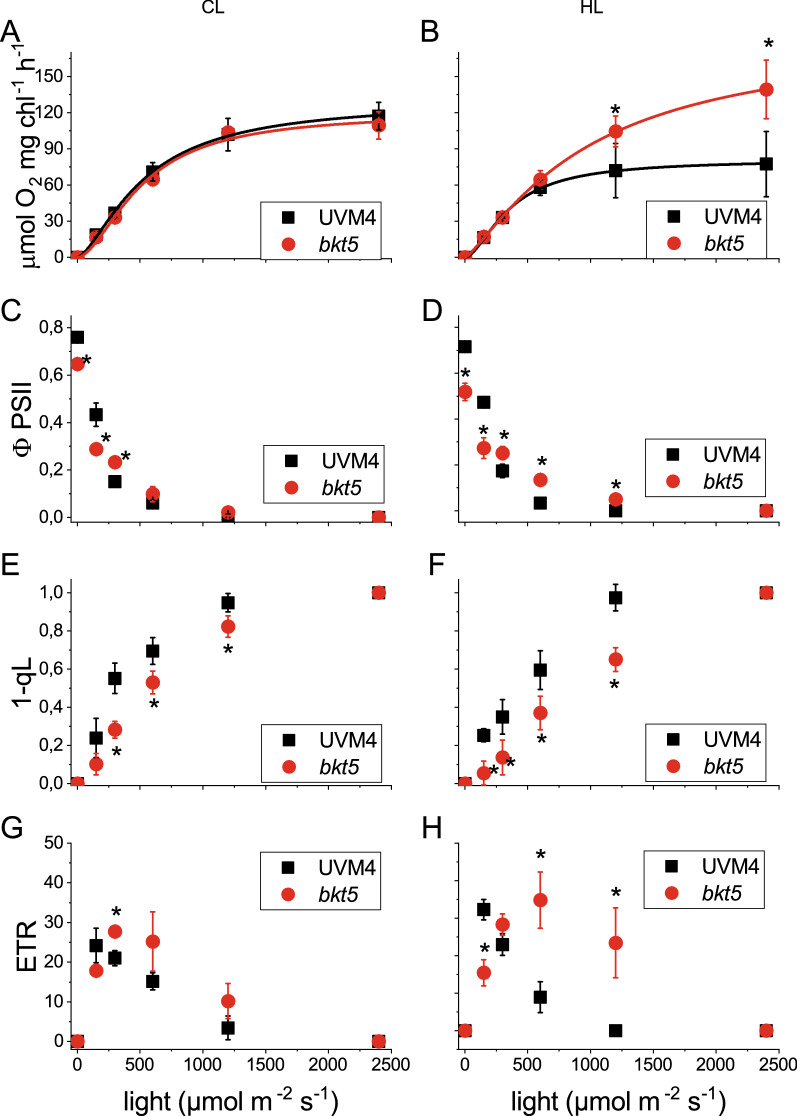
Photosynthetic parameters**.** Photosynthetic parameters of CL and HL-acclimated cells are reported in **A**, **C**, **E**, **G** and **B**, **D**, **F**, **H** panels, respectively. **A**, **B** Net light-dependent O_2_ evolution. **C**, **D** PSII operating efficiency (ΦPSII), **E**, **F** 1‐qL (estimates the fraction of PSII centres with reduced *Q*_A_) and **G**, **H** relative electron transport rate (ETR) at different actinic light intensities for UVM4 (black) and *bkt5* (red). Net photosynthetic rate data were fitted with Hill equation. Error bars are reported as standard deviation (*n* = 3). Values significantly different comparing *bkt5* with UVM4 are reported with * (Student’s *t* test, *P* < 0.05)

Fluorescence induction in dark‐adapted cells [36] revealed a significant decrease of PSII maximum quantum yield (Fv/Fm) in *bkt5* compared to UVM4 (Additional file 1: Table S1) in both CL and HL conditions. To investigate the possible reason at the base of reduced PSII quantum yield in *bkt5* strain, the capacity of LHCII antenna proteins to transfer energy to PSII reaction centre was analysed measuring fluorescence emission spectra at 77 K (Additional file 1: Figure S3). Low-temperature fluorescence spectra of *C. reinhardtii* cells allow to resolve individual fluorescence emission peaks, as the peaks corresponding to PSII-LHCII (686 nm), PSII core (696 nm) and PSI (714 nm) (12); in *bkt5* an additional shoulder at 678 nm was present corresponding to antenna complexes that are not well energetically connected to the reaction centre [37]. In order to evaluate how these changes affect photosynthesis, different photosynthetic parameters were recorded from UVM4 and *bkt5* strains illuminated at increasing light intensities (Fig. 3) [38].

Light-dependent oxygen evolution was initially measured as a direct product of photosynthesis (Fig. 3A, B). In cells acclimated to LL, *bkt5* and UVM4 showed similar maximum light-dependent oxygen production (Pmax), half-saturation light intensity and slope of the linear phase of the oxygen evolution light curve (Additional file 1: Table S3). In HL-acclimated cells the slope of linear increase was still similar but the *bkt5* reached and higher Pmax and the photosynthesis was saturated at a higher light intensity: the half-saturation light intensity was ~ 350 μmol photons m^−2^ s^−1^ or UVM4 and 900 μmol photons m^−2^ s^−1^ for *bkt5*. PSII activity was then analysed measuring fluorescence parameter upon exposure to increasing actinic light (Fig. 3C–F). At 100 μmol photons m^−2^ s^−1^ lower PSII operating efficiency (ΦPSII) and relative electron transport rate (ETR) was measured for *bkt5* compared to UVM4. At higher irradiances *bkt5* showed a value identical to its background or even higher at light intensities in the range of 250–500 μmol photons m^−2^ s^−1^ for samples grown in CL and 250–1.200 μmol photons m^−2^ s^−1^ for cells acclimated to HL. Accordingly, at irradiances higher than 250 μmol photons m^−2^ s^−1^ the fraction of plastoquinone QA reduced, measured as 1-qL and indicative of the saturation of photosynthetic electron transport, was lower in the *bkt5* compared to UVM4 for cells acclimated to either LL or HL. The effect of ketocarotenoids accumulation on PSI was then evaluated measuring kinetics of P700 oxidation at different actinic light (Additional file 1: Figure S4). While in CL-acclimated cells PSI quantum yield (ΦPSI) and ETR_PSI_ were similar between *bkt5* strain and its background, both these parameters were increased at 250 and 1200 μmol photons m^−2^ s^−1^ in *bkt5* strain compared to UVM4 upon HL acclimation. The results obtained for PSII and PSI photosynthetic parameters suggest an improved photosynthetic activity of *bkt5* at high irradiances especially upon HL acclimation.

### Resistance to photoinhibition, role of NPQ and ROS scavenging

**Fig. 4:**
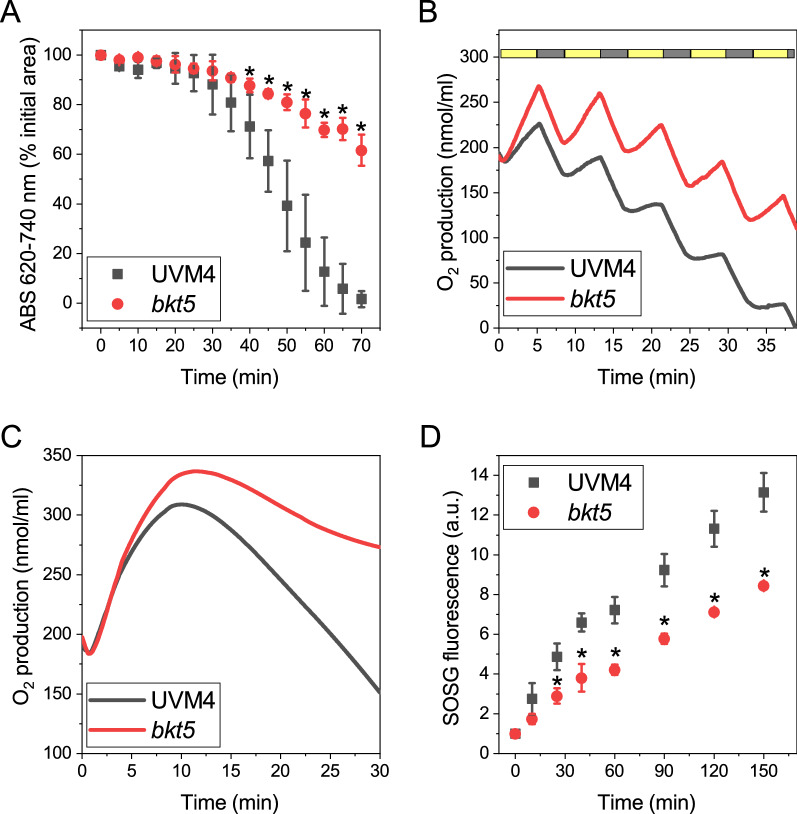
Photooxidation of *bkt5* and UVM4 under light stress. **A** UVM4 (black) and *bkt5* (red) cell suspensions were treated with strong white light (14,000 μmol photons/m^2^/s, 20 °C) and chlorophyll bleaching was evaluated by measuring the absorption area in the region 620–740 nm. **B** Oxygen evolution of UVM4 (black) and *bkt5* (red) cells illuminated with cycles of 5 minutes of white light at 6000 μmol photons m^−2^ s^−1^(yellow bar) and three minutes of dark (black bar). **C** Oxygen evolution during continuous illumination of UVM4 (black) and *bkt5* (red) with white light at 6000 μmol photons m^−2^ s^−1^. **D** Singlet oxygen production of UVM4 (black) and *bkt5* (red) cell suspension, estimated by measuring Singlet Oxygen Sensor Green (SOSG) fluorescence emission (excitation 480 nm, emission 510–540 nm). Cells were illuminated with 2000 μmol photons m^−2^ s^−1^red light at 20 °C. Error bars are reported as standard deviation (*n* = 4). Values significantly different comparing *bkt5* with UVM4 are reported with * (Student’s *t* test, *P* < 0.05)

Under intensive high light, microalgae undergo photooxidative stress leading to ROS production, which causes bleaching of pigments, lipid oxidation and a decrease of photosynthetic efficiency. The improved photosynthetic parameters measured for *bkt5* strain in high light conditions could be related to improved resistance against photoinhibition. For this, Chl bleaching was monitored during illumination with strong high light as an indication of photoinhibition. Cells of UVM4 and *bkt5* strains were illuminated with a strong white light (14.000 μmol photons m^−2^ s^−1^) and every 5 min Qy spectra were quantified (Fig. 4A). A strong reduction in absorbance was detected for UVM4 and after seventy minutes Chl were completely bleached while in *bkt5* only 30% reduction of absorbance was detected at the end of the experiment.

To evaluate the resistance to photoinhibition in terms of photosynthetic activity, light-dependent oxygen production was measured for cells illuminated with strong white light (6.000 μmol photons m^−2^ s^−1^) with cycles of 5 min of light and 3 min of dark compared to 30 min of continuous illumination. In light–dark cycles, oxygen evolution decreased with every cycle due to light-dependent photoinhibition (Fig. 4B), but this effect was much stronger in the case of UVM4 than *bkt5* (Additional file 1: Figure S5). In continuous illumination oxygen evolution increased in the first minutes until a plateau was reached followed by a decrease due to photoinhibition and *bkt5* was more resistant to photoinhibition reaching a higher level of oxygen production followed by a slower decrease compared to UVM4 (Fig. 4C). The reduced Chl/Car ratio observed in *bkt5* (Additional file 1: Table S1) suggests that carotenoids may act as potential filters, specifically absorbing pronounced blue light, which might induce reduced excitation pressure on chlorophylls. In addition, the higher antioxidant activity of ketocarotenoids compared to the natural carotenoids present in UVM4 could contribute to the increased resistance to photoinhibition of *bkt5* strain. The kinetics of singlet oxygen was measured upon exposure to strong red light using a fluorescent dye (singlet oxygen sensor green, SOSG) that increase in fluorescence after interaction with singlet oxygen and can be used to quantify singlet oxygen generation (Flors et al. [68]). It is important to note that using red light, the light-filtering effect due to different carotenoids accumulation in *bkt5* compared to UVM4 is not influencing the measurement. As reported in Fig. 4D, increase in SOSG fluorescence emission could be measured in the case of UVM4, demonstrating an increased antioxidant activity in the case of *bkt5.*

**Fig. 5:**
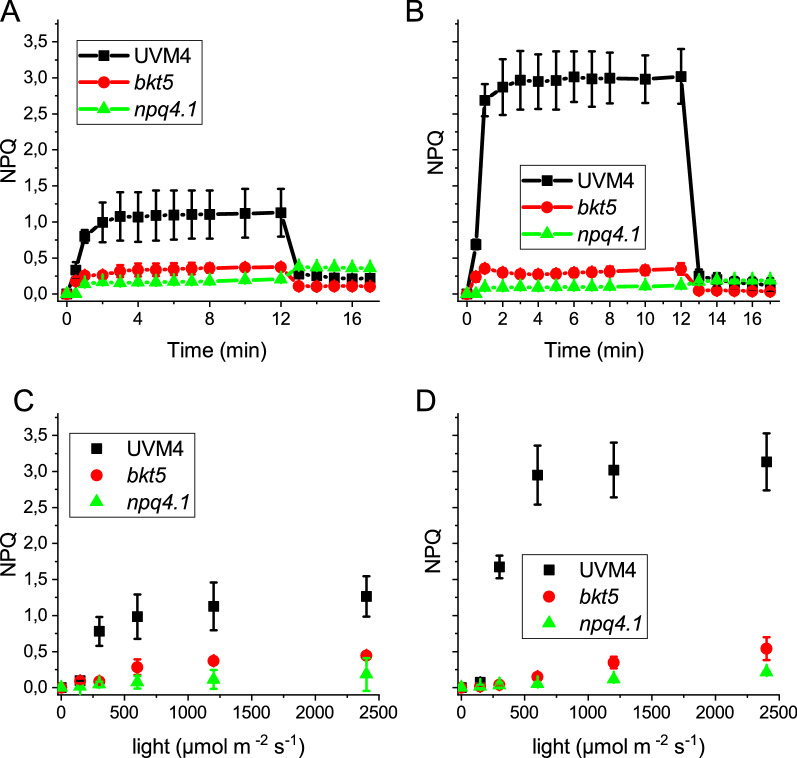
Nonphotochemical quenching (NPQ). **A**, **B** Measurement of NPQ kinetics of UVM4 (black), *bkt5* (red) and *npq4 lhcsr1* (green, indicated as *npq4.1*) cells using actinic lights of 1200 μmol photons m^−2^ s^−1^. **C**, **D** NPQ values after 10 min of illumination with different actinic light intensities. Cells were adapted in control (CL left) of high light (HL right). Error bars are reported as standard deviation (*n* = 4). Values significantly different comparing *bkt5* with UVM4 are reported with * (Student’s t test, *P* < 0.05)

The reduced single oxygen production upon exposure to strong light observed in *bkt5* could be related to increased thermal dissipation of the light energy absorbed due to NPQ activation (9). NPQ induction was measured in *bkt5*, its background UVM4 and, as negative control, in the mutant *npq4 lhcsr1*, unable to activate NPQ due to the absence of the key LHC proteins for NPQ induction, LHCSR1 and LHCSR3 [11, 39]. As reported in Fig. 5, *bkt5* showed a lower NPQ induction compared to cells acclimated to either CL or HL. In all tested conditions *bkt5* and *npq4 lhcsr1* showed similar NPQ which was strongly reduced compared to UVM4 (except for 150 μmol photons m^−2^ s^−1^ when NPQ was essentially not activated even in UVM4) (Additional file 1: Figure S6). NPQ activation is dependent on generation of a proton gradient across the thylakoid membranes and on LHCSR protein accumulation: the strongly reduced NPQ in *bkt5* could be dependent on the impairment of one or both factors [40]. The generation of an electrochemical proton gradient was assessed using ECS‐induced absorbance changes at 520 nm (Additional file 1: Figure S7) [41, 42]. In CL, the ECS measured at the different light intensities was slightly reduced in *bkt5* while at HL the mutant showed a higher ECS with respect to UVM4. The presence of the LHCSR1 and LHCSR3 proteins was checked by immunoblot assay (Fig. 1; Additional file 1: Figure S2). LHCSR1 was present in UVM4 both in CL and HL while LHCSR3 was present only in cells acclimated to HL. On a chlorophyll basis LHCSR1 was slightly reduced in *bkt5* compared to UVM4 in CL, while both LHCSR1 and LHCSR3 were decreased in HL condition. However, it was previously reported that the LHCSR3/PSII ratio is linearly correlated with the NPQ activity of *C. reinhardtii* [10]: on a PSII basis, LHCSR3 level was not significantly different in *bkt5* compared to UVM4 in HL grown cells (Fig. 2). Other LHC proteins that were reported as relevant for NPQ are the monomeric CP26 and CP29 proteins [43], and the subunits of LHCII trimers LHCBM1 [44]: the accumulation of these proteins were similar in *bkt5* and UVM4 or even higher in the case of LHCBM1 and CP29 in HL-acclimated cells (Fig. 1). Thus, the accumulation of LHCSR and other LHC antenna and generation of the proton gradient are likely not at the base of the almost null NPQ phenotype in *bkt5* strain.

It is important to note that NPQ measurements are based on the decrease of the maximum fluorescence emission of PSII upon exposure to actinic light (Fm’) compared to the maximum fluorescence emitted in the dark (Fm). To further investigate if the extremely low NPQ phenotype in *bkt5* was related to a constitutive light-independent quenching of PSII, as recently reported in the case of *C. reinhardtii zep* knock out mutants constitutively accumulating zeaxanthin [45], PSII fluorescence lifetimes were measured in whole cells at 77 K (Additional file 1: Figure S8). At this temperature the photochemical activity is blocked and, by following the emission at 690 nm, it is possible to specifically measure the kinetics of PSII evaluating possible quenching mechanisms. Fluorescence lifetime was recorded from cells previously acclimated to HL, dark-adapted or illuminated one hour with 2400 μmol photons m^−2^ s^−1^ to induce NPQ (Additional file 1: Figure S8). PSII fluorescence lifetimes in dark-adapted samples were similar in the case of UVM4 and *bkt5*. Upon light treatment only UVM4 cells were characterized by a faster PSII fluorescence decay compared to the dark-adapted cells, while in the case of *bkt5* the fluorescence lifetime of dark-adapted or light-treated samples were similar. These results demonstrates that *bkt5* cells remain in an unquenched state even after strong illumination and are consistent with the room temperature pulsed-fluorescence results (Fig. 5) confirming that NPQ mechanism is strongly hampered in *bkt5*.The stronger resistance to photoinhibiton of the *bkt5* was independent from NPQ induction, but likely related to the antioxidant activity of ketocarotenoids.

### Biomass productivity in high light

**Fig. 6:**
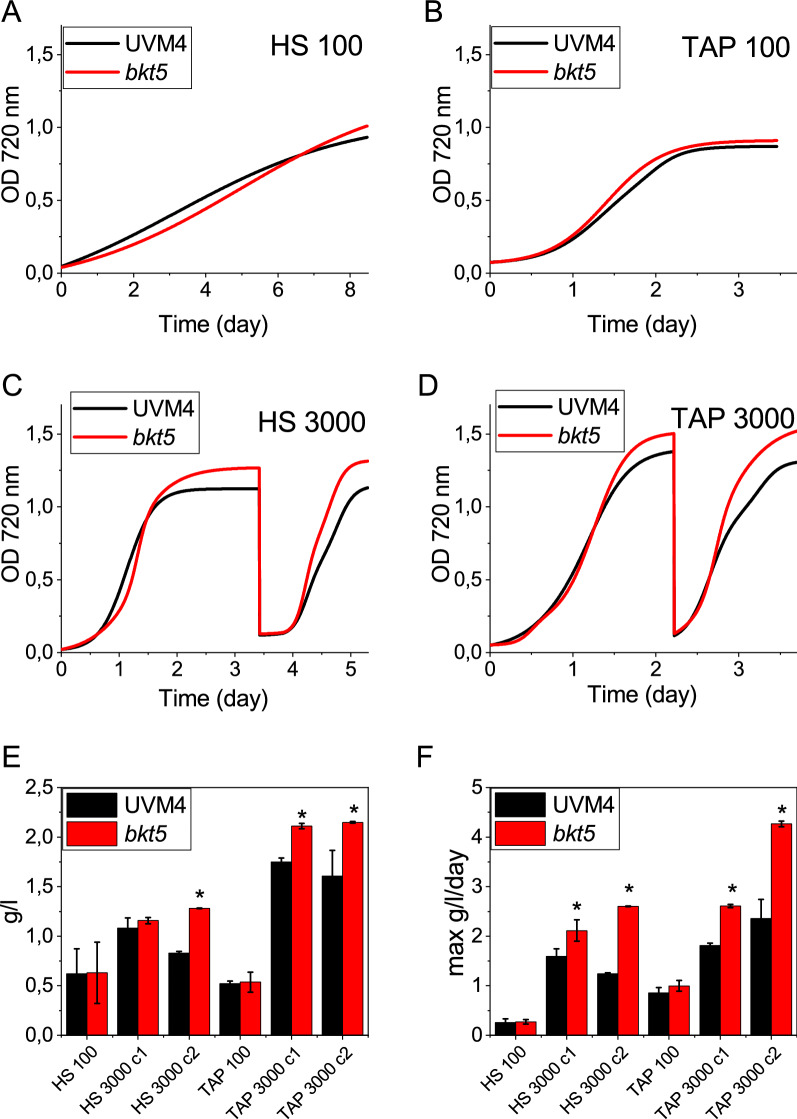
Biomass productivity of UVM4 and *bkt5*. **A**–**D** Growth curves of UVM4 (black) and *bkt5* (red) strains cultivated at 100 and 3000 μmol photons m^−2^ s^−1^ in HS or TAP medium with 3% CO_2_, monitoring OD at 720 nm. In the case of cells grown at 3000 μmol photons m^−2^ s^−1^, when stationary phase was reached, a second growth cycle was performed diluting cells at 0.1 OD. **E**, **F** volumetric biomass (**E**) and maximal productivity (F) in the different growth conditions. For the growth at 3000 μmol photons m^−2^ s^−1^, data were measured at the end of the first (c1) and second cycle (c2). Error bars are reported as standard deviation (*n* = 4). Values significantly different comparing *bkt5* with UVM4 are reported with * (Student’s *t* test, *P* < 0.05)

To investigate the consequences of increased resistance to photoinhibition of *bkt5*, biomass production was evaluated in small-scale photobioreactors (80 ml) in photoautotrophic or mixotrophic condition at 100 μmol photons m^−2^ s^−1^ and 3000 μmol photons m^−2^ s^−1^. At 100 μmol photons m^−2^ s^−1^ in both autotrophic and mixotrophic conditions growth and biomass productivity were similar for UVM4 and *bkt5* strain (Fig. 6). Under 3000 μmol photons m^−2^ s^−1^, *bkt5* grew slower under phototrophic conditions during the first two days but then its kinetic increased reaching a higher maximum daily productivity compared to UVM4. The growth was monitored in a second subsequent cycle to evaluate growth performances of strains already exposed to very high light intensity, observing an increased biomass productivity and a higher biomass concentration at the end of the experiment in the case of *bkt5* strain. In mixotrophic condition at 3000 μmol photons m^−2^ s^−1^, the *bkt5* mutant grown faster, compared to UVM4 from the first cycle.

As reported in Additional file 1: Table S1, *bkt5* and UVM4 were characterized by a different pigment composition: to determine if the increased productivity in *bkt5* compared to UVM4 was related to different self-shading, the transmittance spectra were calculated for both cell cultures considering the absorption spectra of whole cells and the cell density at exponential phase for *bkt5* and UVM4. As reported in Additional file 1: Figure S9, the transmittance spectra were characterized by a reduced transmittance in the 500–560 nm in *bkt5* due to ketocarotenoids absorption, while in the 400–500 nm and 600–700 nm range the transmittance was slightly increased compared to UVM4, due to reduced pigment content per cell in *bkt5*. Considering the light spectrum used for cell cultivation, the 3 cm diameter of the photobioreactors used and the transmittance spectra, the fractions of incident irradiance at different depths in the photobioreactors were calculated for UVM4 and bkt5 strains (Additional file 1: Figure S9): increased 500–560 wavelengths penetration was calculated for UVM4 compared to *bkt5*, which however was characterized by slightly increased penetration of 400–500 and 600–700 nm wavelengths in the latter. Considering that blue (400–500 mn) and red (600–700 nm) lights are the main spectral range triggering photosynthesis, we cannot exclude that increased productivity of *bkt5* could be at least partially related to improved penetration of this wavelengths compared to its background strain, even if this likely not the main reason for the increased productivity observed.

**Fig. 7:**
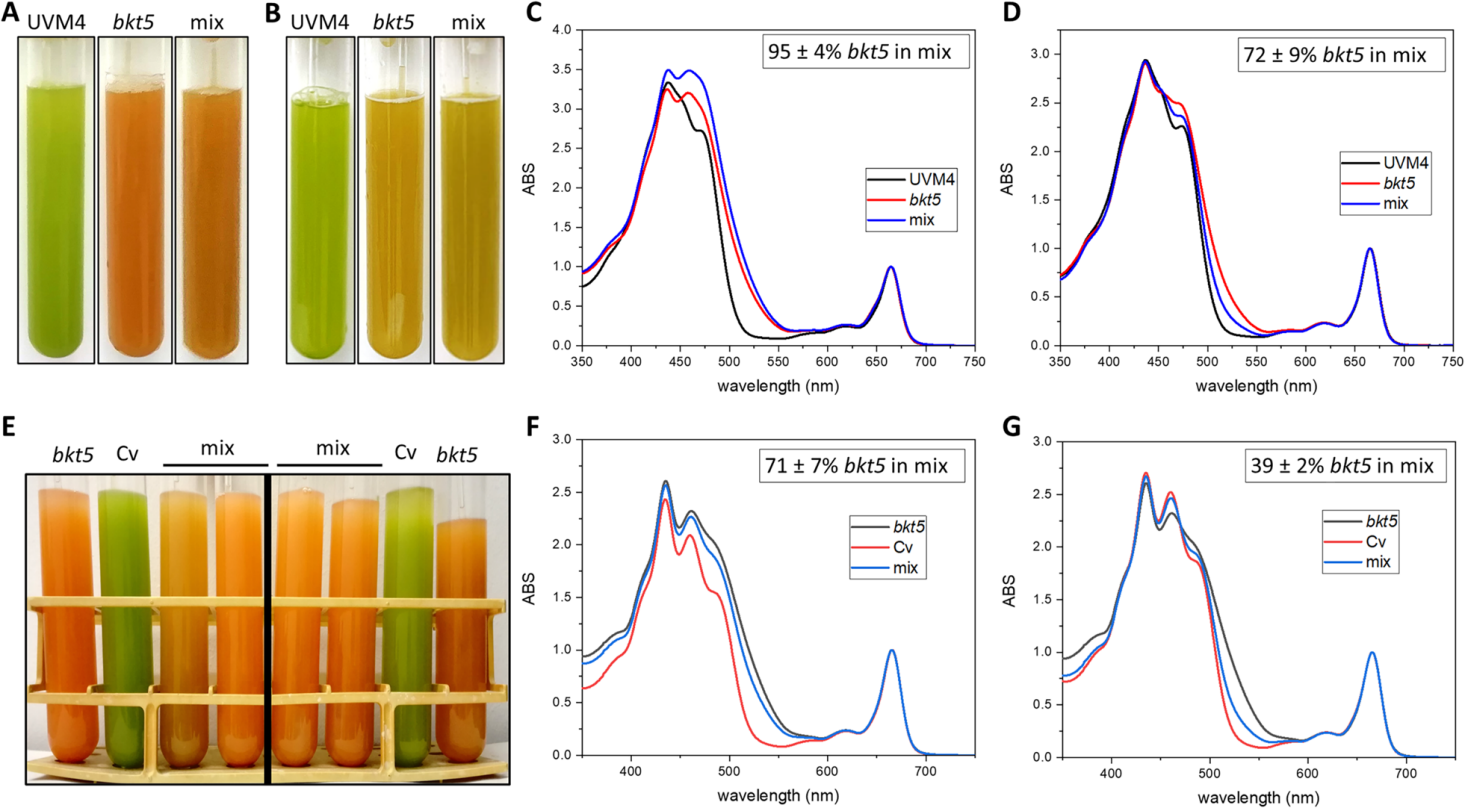
Competitive growth of bkt5 with UVM4 and Chlorella vulgaris. **A**, **B** Picture taken at the end of the growth in the case of cells grown in TAP (**A**) of HS (**B**) media of UVM4 (black), *bkt5* (red) or a mix of the two genotypes in equal amount (blue), at 3000 μmol photons m^−2^ s^−1^ as reported in Fig. 6. **C**, **D** Spectra of the pigments extracted at end of the growth in TAP (**C**) of HS (**D**) experiment; spectra are normalized to the Qy absorbance. The inset shows the calculated percentage of *bkt5* cells inside the mix tube. (**E**) Picture taken, at the end of the growth at 3000 μmol photons m^−2^ s^−1^ of *bkt5*, *C. vulgaris* (Cv) and a mix of the two genotypes. Tubes on the left side of the black line show the result of competition test starting from the same number of cells. Tubes on the right side show the result of mix starting from the same chlorophyll amount. **F**, **G** Spectra of the pigments extracted from the tubes of *bkt5* (black), Cv (red) and the mix (blue) at the end of experiment starting from the same number of cells (**F**) or the same Chl amount (**G**). Spectra are normalized to the Qy absorbance. Insets show the calculated percentage of *bkt5* cells inside the mix tube

To further confirm this enhanced productivity of *bkt5* strain in high light, a competitive growth was performed in both autotrophy and mixotrophy conditions (Fig. 7A–D, Additional file 1: Figure S10). An equal amount of UVM4 and *bkt5* cells was added to same photobioreactor and illuminated with 3000 μmol photons m^−2^ s^−1^: at the end of the cultivation the strain composition within the photobioreactor was analysed to determine which genotype was more accumulated. Astaxanthin, with its shifted absorption, were present only in the *bkt5* and could be used to determine the relative abundance of the two genotypes. Preliminarily, a titration curve reporting the genotype percentage as a function of astaxanthin absorbance was obtained by mixing different amount of UVM4 and *bkt5* strains grown separately at 3000 μmol photons m^−2^ s^−1^ and extracting pigments to determine astaxanthin absorption by deconvolution of pigments absorption spectra (Additional file 1: Figure S11A). In TAP medium at the end of the cultivation more than  ~ 90% of the cells in the photobioreactor were *bkt5* cells (Fig. 7C). The competition test was then repeated in HS medium where again *bkt5* cells become dominant over UVM4 cells, with  ~ 75% of cells at the end of the experiment in the photobioreactor being *bkt* cells (Fig. 7D).

A similar experiment was then repeated to compare the performance of *C. reinhardtii bkt5* strain with *Chlorella vulgaris* (Fig. 7E–G, Additional file 1: Figure S10)*,* a microalgal strain, well known for its capacity to tolerate higher light intensities and to grow faster than *C. reinhardtii* [46–48]. The test was performed mixing an equal amount of *bkt5* and *C. vulgaris* cells (5*10^5^ cell/ml each) and growing the cell mixture at 3000 μmol photons m^−2^ s^−1^. As for the previous tests, a calibration curve of different microalgae percentage vs ketocarotenoid content was obtained (Additional file 1: Figure S11B). Surprisingly, *bkt5* strain showed a higher growth than *C. vulgaris* and at the end of the experiment, the percentage of *bkt5* cells in the mix tubes was 87 ± 1%. It should note that the preparation of the cell mixture at the beginning of the experiment using the same cell number could be subject to some bias because *C. vulgaris* cells are smaller than *C. reinhardtii* ones (~ 1 vs 10 µm). The competition test between *C. reinhardtii bkt5* and *C. vulgaris* cells was thus repeated mixing the two strains to have the same Chl content for the two species in the photobioreactor*.* Also in this case, *bkt5* strain become dominant over *C. vulgaris* with more than 90% of the cells being *bkt5* cells at the end of the experiment. These competition tests, clearly point out how the presence of astaxanthin in *C. reinhardtii* increases its resistance to ROS and photoinhibiton and improve the performance in photobioreactor in high light and biomass accumulation.

## Discussion

The expression of an active BKT enzyme profoundly changed the specific pigment accumulation and composition in *C. reinhardtii*. In *bkt5* more than 50% of carotenoids were converted into ketocarotenoids, ~ 74% and  ~ 56% of which being astaxanthin for cells grown, respectively, in CL or HL. Moreover, Chl/Car ratios were decreased in both CL and HL conditions indicating, respectively, a ~ 17% and  ~ 20% increase in total carotenoids accumulation on a Chl basis (Additional file 1: Table S1, Table S2). It is important to note that overall pigment content per cell in *bkt5* was reduced, with a decrease in both Chl and carotenoid accumulation on a cell basis (Additional file 1: Table S1). However, the increased carotenoids content per Chl implies an increased availability of carotenoids as photoprotective agent for the Chl accumulated in the thylakoid membranes. The altered carotenoids composition with ketocarotenoids partially substituting xanthophylls and carotenes (Additional file 1: Figure S1) destabilizes the photosynthetic complexes affecting mainly the trimerization state of LHCII, PSII core and PSI (Fig. 1). The PSII core complex was more affected by the alteration in carotenoids: in *bkt5* the accumulation of PSII complexes was greatly impaired while the assembly of PSI was less sensitive to carotenoid availability [49, 50]. The lack of appropriate carotenoids could result in a reduced efficiency in PSII assembly. Ketocarotenoids were found distributed throughout the overall fractions in sucrose gradient which could be related to contamination due to high accumulation of astaxanthin in the thylakoids. Alternatively, partial substitution of xanthophylls and carotenes by ketocarotenoids cannot be excluded, but the binding of these carotenoids to photosynthetic complexes need further work to be verified. It is likely that the molecular structure of astaxanthin (e.g., carboxylation of β-rings) does not fit with photosynthetic proteins or that their interaction is weaker than endogenous xanthophylls and it is disrupted by detergent solubilization. Accordingly, the distribution of the astaxanthin in thylakoid membranes (Additional file 1: Figure S1A) showed that the main part of the ketocarotenoid was not bound to the photosynthetic complexes but free in the membranes. Similar consequences were also observed in *A. thaliana* and tobacco plants engineered to accumulate astaxanthin [31, 51, 52]. In the case of *H. lacustris*, astaxanthin is mainly accumulated outside the chloroplast, but even in this case the small fraction of this ketocarotenoid detected in the plastids is mainly free in the thylakoid membranes [26].

The changes in carotenoids composition and partial destabilization of the photosynthetic complexes affect the photosynthetic performance in *bkt5* (Fig. 3). The Fv/Fm was reduced in *bkt5* by ~ 18% while, upon exposure to actinic lights, ΦPSII, ETR and 1-qL were reduced at 100 μmol photons m^−2^ s^−1^ (Fig. 3). However, the reduced PSII efficiency had almost negligible effect on the growth of *bkt5* at this light intensity (Fig. 6). This discrepancy between the negative impact of BKT expression on photosynthetic parameters at low light and comparable biomass productivity for *bkt5* and UVM4 strains under autotrophic or mixotrophic conditions, could be explained with the “pale” phenotype of *bkt5*. Decreasing the chlorophyll content of microalgae cells is a strategy successfully applied to increase solar energy conversion efficiency enhancing light penetration inside algal culture and minimizing feedback energy dissipation [53, 54]. As reported in Additional file 1: Figure S9, the reduction of both Chl and carotenoids per cell content in *bkt5* caused a slightly enhanced penetration of blue and red light*,* possibly compensating the reduced efficiency of the PSII, even if this speculation requires further evidences to be supported. We cannot even exclude that the reduced penetration of the 500–560 nm wavelengths in *bkt5* culture might contribute to some extent to the increased photoresistance of this strain, involving some signal transmission pattern related to some specific photoreceptors as channel rhodopsins [55, 56], which perceives light at these wavelengths. At higher irradiances, PSI and PSII photosynthetic parameters as ΦPSI, ΦPSII, ETR and ETR_PSI_, were similar or even higher in *bkt5* compared to UVM4. Moreover, the photosynthetic rate, measured as oxygen evolution, showed a higher *P*_max_ and a higher half-saturation intensity (Additional file 1: Table S3). These results indicate that the negative effect on the stability of photosynthetic complexes was compensated by an increased resistance to high light. Accordingly, the biomass productivity was strongly enhanced in *bkt5* when grown at 3000 μmol photons m^−2^ s^−1^ either in autotrophy or in mixotrophy (Fig. 6). This effect is particularly evident from growth curves and productivity of the second cycles of growth when the cell were already exposed to high illumination and ketocarotenoid content was increased (Fig. 6E, F). Moreover, *bkt5* outcompeted its parental strain when competitively co-cultured in the same photobioreactor (Fig. 7). Surprisingly, *bkt5* strain was able to become dominant even in a competition test with *C. vulgaris*, one of the green alga species more considered for industrial application due its fast-growing phenotype (Fig. 7).

These results were quite surprising since in land plant expressing BKT, there were no reported evidence for an increased productivity while in some cases the transgenic plant showed a hampered growth compared to the control [31, 51, 52]. This is likely related to the simple structure of unicellular organism like microalgae that can cope better with the severe changes in carotenoids composition. It is interesting to note that even in the case of astaxanthin-rich *H. lacustris* cysts an increase in P_max_ on a Chl basis was observed compared to the “green” vegetative cells [57]. In *C. reinhardtii bkt5* astaxanthin accumulated constitutively during growth phase [28], whereas natively it requires environmental stress-related activation in *H. lacustris*, which simultaneously induces profound changes of cellular structure and a shift to the aplanospore phase [25].

Improved biomass productivity in *C. reinhardtii* could be mediated by the increased light penetration due to reduced chlorophyll contents, although application of higher light intensities should compensate diffusion limitations and ensures sufficient light energy supply in *C. reinhardtii* cultures. But it is likely that increased resistance to photoinhibition in *bkt5* is assisting in tolerating high light, as measured from chlorophyll bleaching, oxygen evolution and singlet oxygen production upon exposure to photo-inhibiting irradiances (Fig. 4).

The main mechanism for protection from high light is the NPQ that dissipates the excessive light energy as heat. A higher resistance to photoinhibition could be due to an improved quenching mechanism, but this was not the case of *bkt5* where a strong reduction in NPQ was evident in cells acclimated either to CL or HL. Activation of NPQ in *C. reinhardtii* is dependent on generation of protonic gradient in the thylakoid lumen and accumulation of antenna protein LHCSR1 and LHCSR3 [11, 15, 40]. ECS measurements demonstrated that proton transport into the lumen was similar or even higher in *bkt5* strains compared to its background strain. In *bkt5* LHCSR1 is present both in CL and HL even if its level is reduced compared to UVM4. The reduction of LHCSR1 is likely not the cause for the impaired NPQ since LHCSR3 is primarily responsible for quenching activation in high light-acclimated cells [11] and *C. reinhardtii* mutant without LHCSR1 showed the same NPQ level as wild type [12]. LHCSR3 is synthetized in *bkt5* only in HL: on chlorophyll basis LHCSR3 was reduced in *bkt5* but on a PSII basis, there was no significant difference between the two genotypes (Fig. 2): because it was reported that the LHCSR3/PSII ratio is linearly correlated with the NPQ capability of *C. reinhardtii* [10], the different LHCSR3 expression on a chlorophyll basis is likely not sufficient to explain the NPQ phenotype of *bkt5* strain. However, it is important to note that while LHCSR3/PSII ratio is essentially unchanged, *bkt5* exhibits an increased LHCII/PSII ratio: being the energy absorbed by LHCII one of the main targets of NPQ, we cannot exclude that the reduced LHCSR3/LHCII ratio in *bkt5* could at least partially contribute to the reduced NPQ phenotype in this engineered strain compared to UVM4. Astaxanthin accumulation in tobacco plants was previously reported to induce LHCII antenna proteins into a light-independent quenched state [58]. However, this possible constitutive quenching mechanism had a minor role in *C. reinhardtii bkt5* because 77 K fluorescence measurements on dark or light-treated whole cells demonstrated that PSII in *bkt5* cells is not in a constitutive quenching state (Additional file 1: Figure S8). The reduction of NPQ was more probably due to the changes in carotenoids compositions; mutants with altered xanthophylls and carotenes composition showed a reduction of NPQ both in microalgae and land plant [59, 60]. However, independently from the reason for its reduction, the NPQ mechanisms are not involved in the increased resistance to strong irradiances observed in *bkt5*.

The increased resistance to high light of *bkt5* strain must then directly depend on astaxanthin and ketocarotenoids accumulation. Astaxanthin could probably exert this role with different mechanisms. (i) The increased carotenoid contents per chlorophyll observed in *bkt5*, mainly related to ketocarotenoids accumulation, makes astaxanthin as a “sunscreen” for chlorophylls, reducing the penetration of blue light into the thylakoids, thereby reducing excessive light absorption by photosystems [61]. The reduced singlet oxygen production, measured by SOSG fluorescence, in *bkt5* compared to UVM4 upon red light only exposure demonstrates, however, that the light-filtering effect of ketocarotenoids in the 500–560 nm (Additional file 1: Figure S9) is not the only mechanism contributing to increased photoprotection in *bkt5*. (ii) Astaxanthin is one of the most powerful antioxidant molecules and can scavenge ROS and could act as a barrier that prevents lipids, pigments, and photosynthetic complexes oxidation. Astaxanthin showed an antioxidant activity against singlet oxygen far higher than β-carotene [23, 62]. According to this hypothesis, singlet oxygen was detected in far higher amount in UVM4 compared to the strain that accumulates astaxanthin (Fig. 4C). This result is different from what was observed in *H. lacustris* where astaxanthin accumulating “red” cells showed a reduced Chl bleaching but the same amount of singlet oxygen generation. This difference could depend on the fact that in *bkt5* ketocarotenoids are accumulated in the chloroplast, the main site of light-dependent ROS production, while in *H. Lacustris* astaxanthin is present in lipid droplets in the cytosol, having a major role as a light filter. (iiii) The extensive accumulation of astaxanthin could be an additional way to use the high level of reducing power generated during continuous illumination regenerating NADP^+^ as electron acceptor for the photosynthetic apparatus, mitigating the risk of saturation of the electron transport chain [63]. All these aspects can contribute to the higher resistance of *bkt5* in high light. It is important to consider a possible astaxanthin dose response for the high light tolerance in *bkt* strains: *bkt5* strain herein investigated was selected for having high astaxanthin and accumulation (up to 2.5 mg/g dry weight), but further work is required to identify the best astaxanthin and ketocarotenoid cell concentration to improve biomass production in high light conditions.

## Conclusions

The results presented here demonstrate that tailored pigments composition and addition of ketocarotenoids with higher antioxidant activity is a viable strategy to induce high light resistance and improve photosynthetic efficiency of *C. reinhardtii* and likely other green algae. Astaxanthin accumulated in engineered *C. reinhardtii bkt5* strain was found mainly free in the thylakoid membranes or possibly bound to both PSI and PSII. The changes in pigments composition affect the photosynthetic machinery in low light condition, but the presence of ketocarotenoids gives a striking advantage in constant high light. Very high light (up to 3000 μmol photons m^−2^ s^−1^) natively occurs in outdoor cultivation strategies in extreme environments but induced strong photoinhibition in wild-type *C. reinhardtii* reducing overall efficiency. Engineered astaxanthin accumulation significantly improved growth under high light, reduced photoinhibition and could be proposed as a novel strategy to outperform growth of competing microalgae or cyanobacteria strains.

## Methods

### Algal cultivation and growth analysis

*Chlamydomonas reinhardtii* strain UVM4 [32] and *Chlorella vulgaris* 211/11P (Culture Collection of Algae at Goettingen University CCAP 211/11P strain) were maintained on TAP agar plates or in liquid shake flasks at 25 °C with 100 μmol photons m^−2^ s^−1^ of continuous white light. Acclimation to control light (CL) or high light (HL) was performed in flasks in HS medium, respectively, at 100 and 600 μmol photons m^−2^ s^−1^. Growth tests were performed using Multi-Cultivator MC-1000 (Photon Systems Instruments) with continuous monitoring of optical density (OD) at 680 and 720 nm. Temperature was maintained at 25 °C, while light intensities varied between 100 and 3000 μmol photons m^−2^ s^−1^ as described at the respective experiment. Tris-acetate phosphate (TAP) or high-salt (HS) media were used for mixotrophic or photoautotrophic conditions, respectively [33]. Cell densities were measured using Countess II FL Automated Cell Counter (Thermo Fisher Scientific). Dry biomass was evaluated by overnight lyophilization of washed cell pellets and gravimetric determination.

### Membrane preparation and fractionation

Thylakoid membranes were isolated and fractionated as previously described [64].

### Spectroscopy and pigments analyses

Pigments were extracted from intact cells and gradient fractions, using 80% acetone buffered with Na_2_CO_3_. Absorption spectra were fitted with the different pigment absorption spectral forms [28]. Pigment extracts were analysed by high-performance liquid chromatography as described previously for *bkt* strains [28]. Absorption spectra were measured using a Jasco V-750 UV/VIS spectrophotometer. Steady-state 77 K fluorescence emission spectra were, on cell frozen in liquid nitrogen, using a BeamBio custom device equipped with USB2000 Ocean Optics spectrometer (Ocean Optics). Time-resolved fluorescence emissions at 77 K were measured as previously reported [65]. In particular, cells after dark adaptation or after 60 min of illumination at 1500 µmol photons/m^2^/s of light were frozen in liquid nitrogen. Chronos BH ISS Photon Counting instrument with picosecond laser excitation at 447 nm operating at 50 MHz was used to measure fluorescence decay kinetics. Fluorescence emissions were recorded at 690 nm with 4 nm bandwidth.

### SDS-PAGE electrophoresis and immunoblotting

SDS-PAGE analysis was performed using the Tris–Tricine buffer system [66]. Thylakoid samples were loaded for each sample and electroblotted on nitrocellulose membranes, and proteins were quantified with an alkaline phosphatase-conjugated antibody system: αPSAA (AS06 172), αCP43 (AS11 1787), αCP29 (AS04 045), αCP26 (AS09 407), αLHCII (AS01 003), αLHCSR1 (AS14 2819), αLHCSR3 (AS14 2766), antibodies were purchased from Agrisera.

### Photosynthetic parameters and NPQ measurements

Photosynthetic parameters ΦPSII, qL, electron transport rate (ETR), and NPQ were characterized by measuring with a DUAL-PAM-100 fluorimeter (Heinz–Walz) chlorophyll fluorescence of intact cells, at room temperature in a 1 × 1 cm cuvette mixed by magnetic stirring. ΦPSII, qL, and ETR were measured upon 20 min of illumination as previously reported [38, 67]. NPQ measurements were performed on dark-adapted intact cells, with a saturating light of 4000 μmol photons m^−2^ s^−1^ and different actinic lights indicated. ΦPSI and PSI ETR were measured following the transient absorption at 830 nm upon exposure to different actinic lights. Proton motive force upon exposure to different light intensities was measured by electrochromic shift (ECS) with MultispeQ V2.0 (PhotosynQ) (Kuhlgert et al. 2016).

### Oxygen evolution

The oxygen evolution activity of the cultures was measured at 25 °C with a Clark-type O_2_ electrode (Hansatech), during illumination with light from a halogen lamp (Schott). Measurements were performed in 1 ml cell suspension concentrated at 5 × 10^6^ cell ml^−1^.

### Determination of the sensitivity to photooxidative stress

The photobleaching kinetics of cells was measured using an actinic light of 14,000 μmol photons m^−2^ s^−1^ in 1 ml cell suspension at 20 °C. The initial cellular concentration was adjusted to have comparable absorbance at 620–740 nm for all samples. The absorption in the same wavelength range (620–740 nm) was measured to follow Chl bleaching upon light exposure. Singlet oxygen production was measured using the Singlet Oxygen Sensor Green (SOSG), a fluorescent probe that increases the intensity of its emission in the presence of this ROS (Flors et al. [68]). Cell suspension, incubated with SOSG, were illuminated with red light at 2000 μmol photons m^−2^ s^−1^ at 20 °C. Singlet oxygen production was measured as increased fluorescence emission of SOSG with respect to the initial value (excitation 480 nm, emission 510–540 nm). The initial cellular concentration was set to have the same absorbance area in the Qy for all the samples.

## Supplementary Information

**Additional file1**: **Figure S1**: Ketocarotenoid distribution in *bkt5* sucrose gradient fractions. **Figure.S2**: Photosynthetic subunit protein content on a chlorophyll basis*.* **Figure S3**: 77 K fluorescence emission spectra of UVM4 and *bkt5* whole cells. **Figure S4**: PSI electron flow. **Figure S5**: Rate of oxygen evolution during high light stress. **Figure S6**: Nonphotochemical quenching (NPQ) at different light intensities. **Figure S7**: Estimation of total proton motive force. **Figure S8**: Time resolved fluorescence emission of whole cells at 77 K. **Figure S9**: Transmittance and light penetration in photobioreactors. **Figure S10**: Growth curves of UVM4, *bkt5* and *Chlorella vulgaris* in competitive growth tests. **Figure S11**: Titration curve of *bkt5* cells in mixture with UVM4 and *C. vulgaris* cells. **Table S1**: Pigment content and Fv/Fm of UVM4 and *bkt5*. **Table S2**: Carotenoids content of UVM4 and *bkt5*. **Table S3**: Photosynthesis and respiration rates

## Data Availability

All data generated or analysed during this study are included in this published article [and its supplementary information files.
