# Supplementary material for: Engineering astaxanthin accumulation reduces photoinhibition and increases biomass productivity under high light in Chlamydomonas reinhardtii
Source: Biotechnol Biofuels Bioprod. 2022 Jul 11;15:77. doi: 10.1186/s13068-022-02173-3 (PMC9277849; doi:10.1186/s13068-022-02173-3)
Supplement: Supplementary file 1 — Additional file1: Figure S1: Ketocarotenoid distribution in bkt5 sucrose gradient fractions. Figure.S2: Photosynthetic subunit protein content on a chlorophyll basis. Figure S3: 77 K fluorescence emission spectra of UVM4 and bkt5 whole cells. Figure S4: PSI electron flow. Figure S5: Rate of oxygen evolution during high light stress. Figure S6: Nonphotochemical quenching (NPQ) at different light intensities. Figure S7: Estimation of total proton motive force. Figure S8: Time resolved fluorescence emission of whole cells at 77 K. Figure S9: Transmittance and light penetration in photobioreactors. Figure S10: Growth curves of UVM4, bkt5 and Chlorella vulgaris in competitive growth tests. Figure S11: Titration curve of bkt5 cells in mixture with UVM4 and C. vulgaris cells. Table S1: Pigment content and Fv/Fm of UVM4 and bkt5. Table S2: Carotenoids content of UVM4 and bkt5. Table S3: Photosynthesis and respiration rates [file 13068_2022_2173_MOESM1_ESM.pdf]

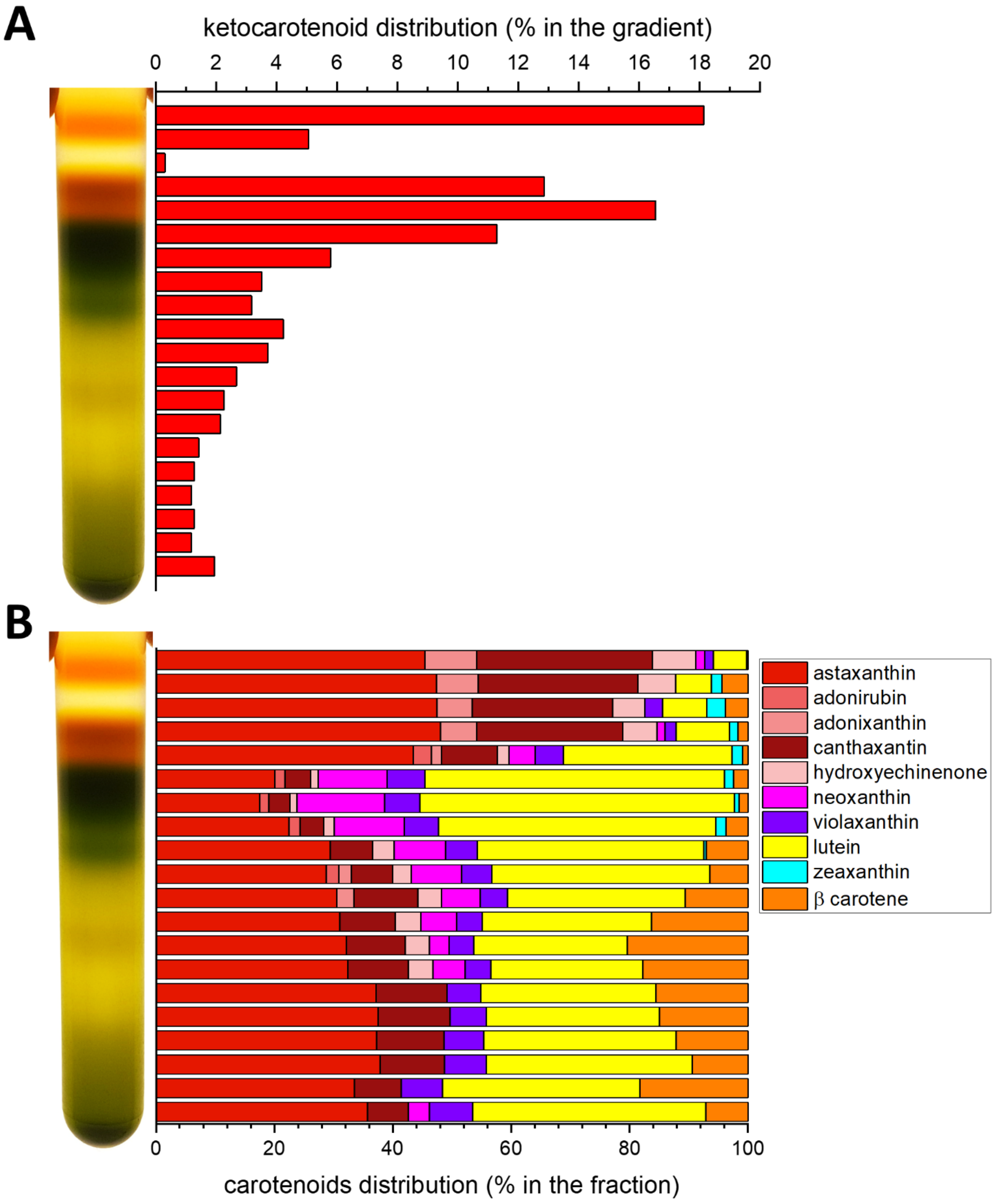

Figure S1. **Ketocarotenoid distribution in *bkt5* sucrose gradient fractions.** Thylakoid membranes isolated from *bkt5* cells were solubilized with 0.8% α-DM and fractionated by ultracentrifugation in sucrose gradient. (A) Distribution in percentage of the total ketocarotenoids in the gradient fractions. (B) Carotenoids composition in percentage of each fraction.

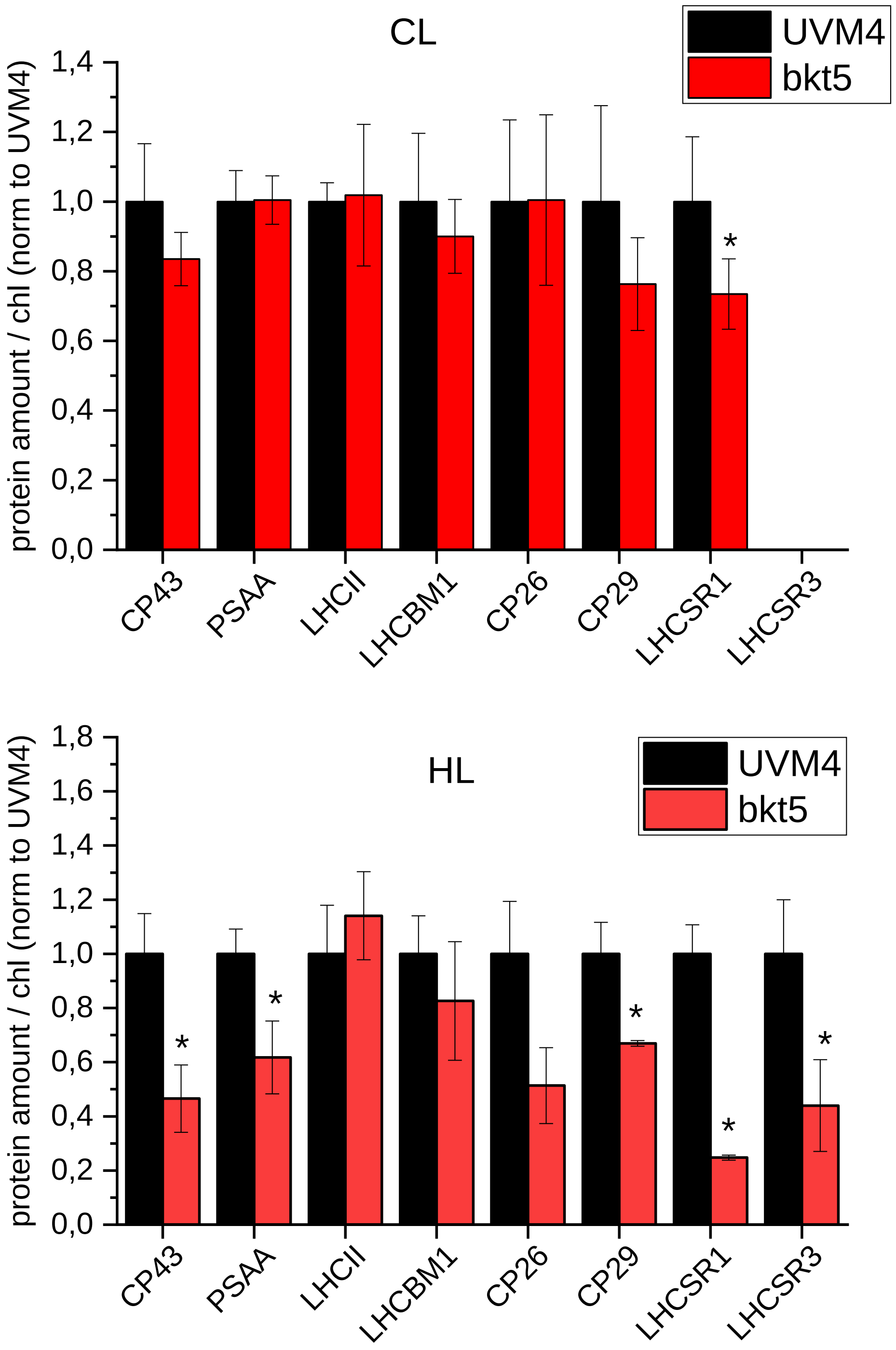

Figure S2. **Photosynthetic subunit protein content on a chlorophyll basis.***)* Densitometric analysis of immunoblotting results reported in Figure 2. Data are reported on a chlorophyll basis and set to 1 in the case of UVM4. Error bars are reported as standard deviation (n = 4). Values significantly different comparing *bkt5* with UVM4 are reported with * (Student's t test, P < 0.05).

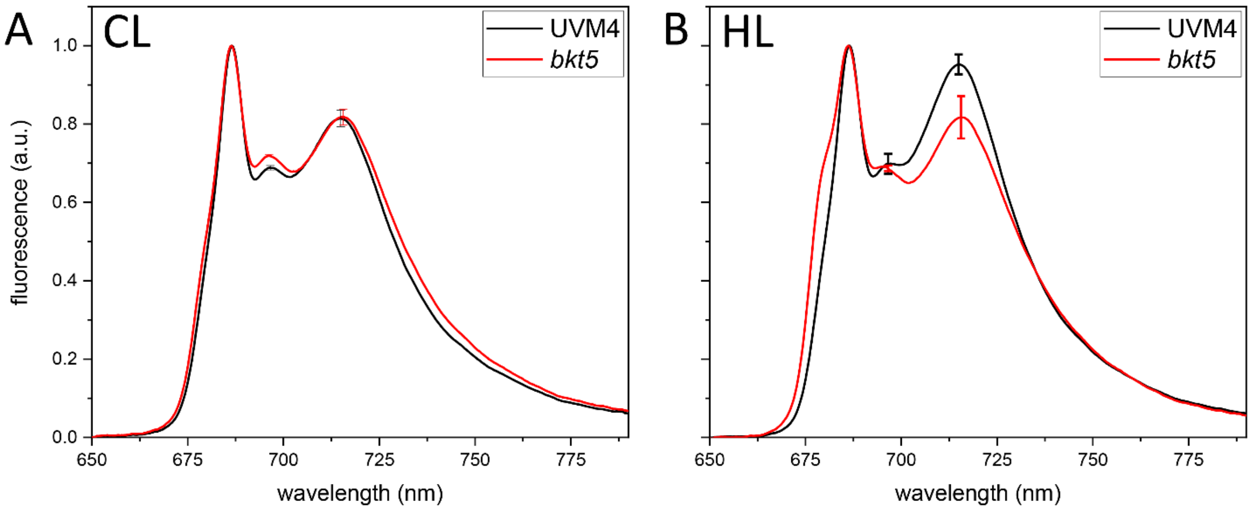

Figure S3. 77K fluorescence emission spectra of UVM4 and *bkt5* whole cells. 77 K fluorescence emission spectra of UVM4 (red) and *bkt5* (black) cells adapted in control light (CL, 100 µmol photons m^{-2} s^{-1}) or high light (HL, 600 µmol photons m^{-2} s^{-1}). Sample were excited at 440 nm and normalized to maximum in PSII emission. Error bars are reported as standard deviation (n = 3).

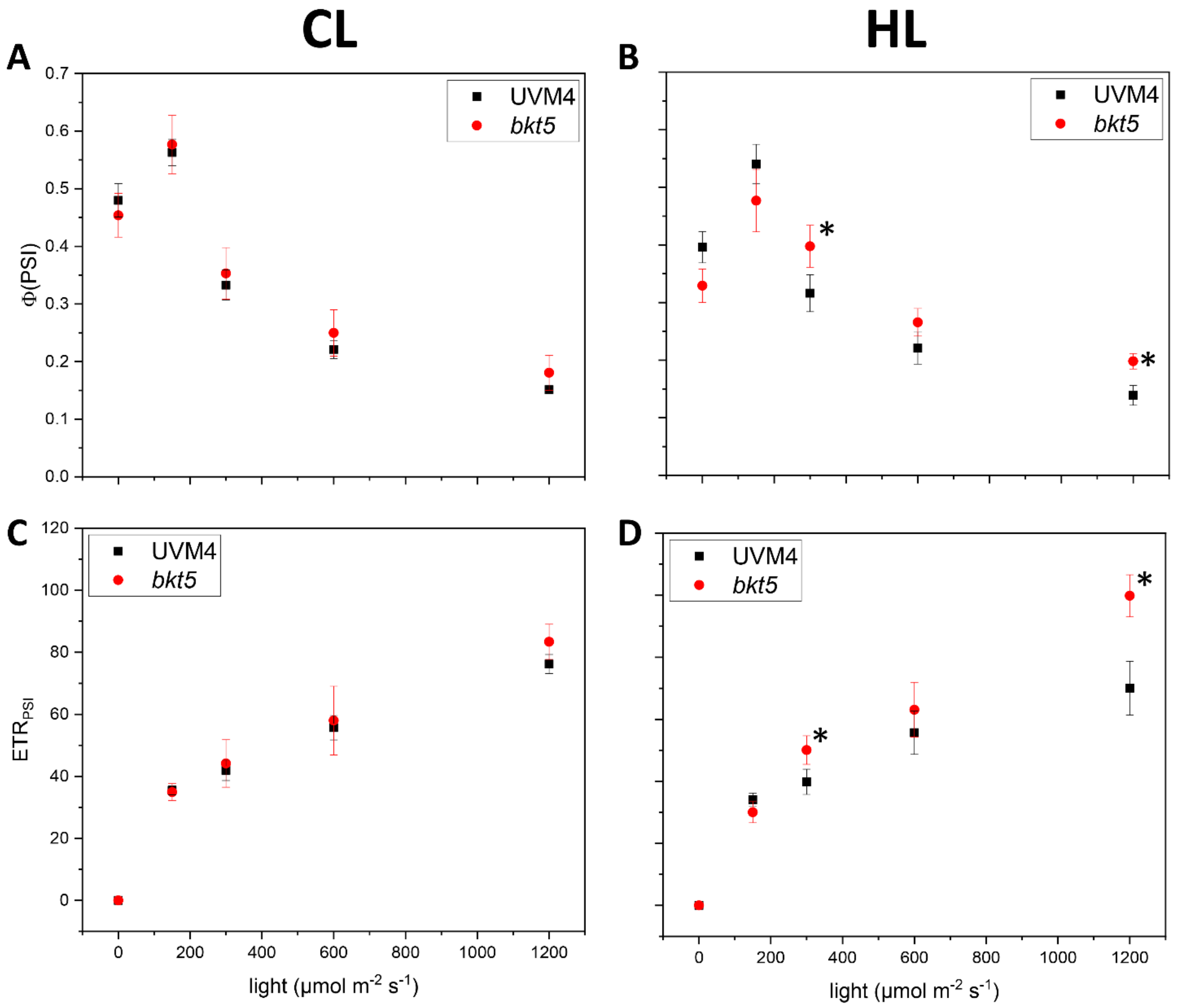

Figure S4. PSI electron flow. PSI quantum yield (ΦPSI,A,B) and PSI electron transport rate (ETR_{PSI},C;D) upon exposure to different light intensities. All measurement were performed on UVM4 (black) and *bkt5* (red) cells adapted to control (CL, left) and high light (HL, right). Data are expressed as mean ± SD (n = 3). * indicates *bkt5* values that are significantly different (Student's test, P < 0.05) from UVM4.

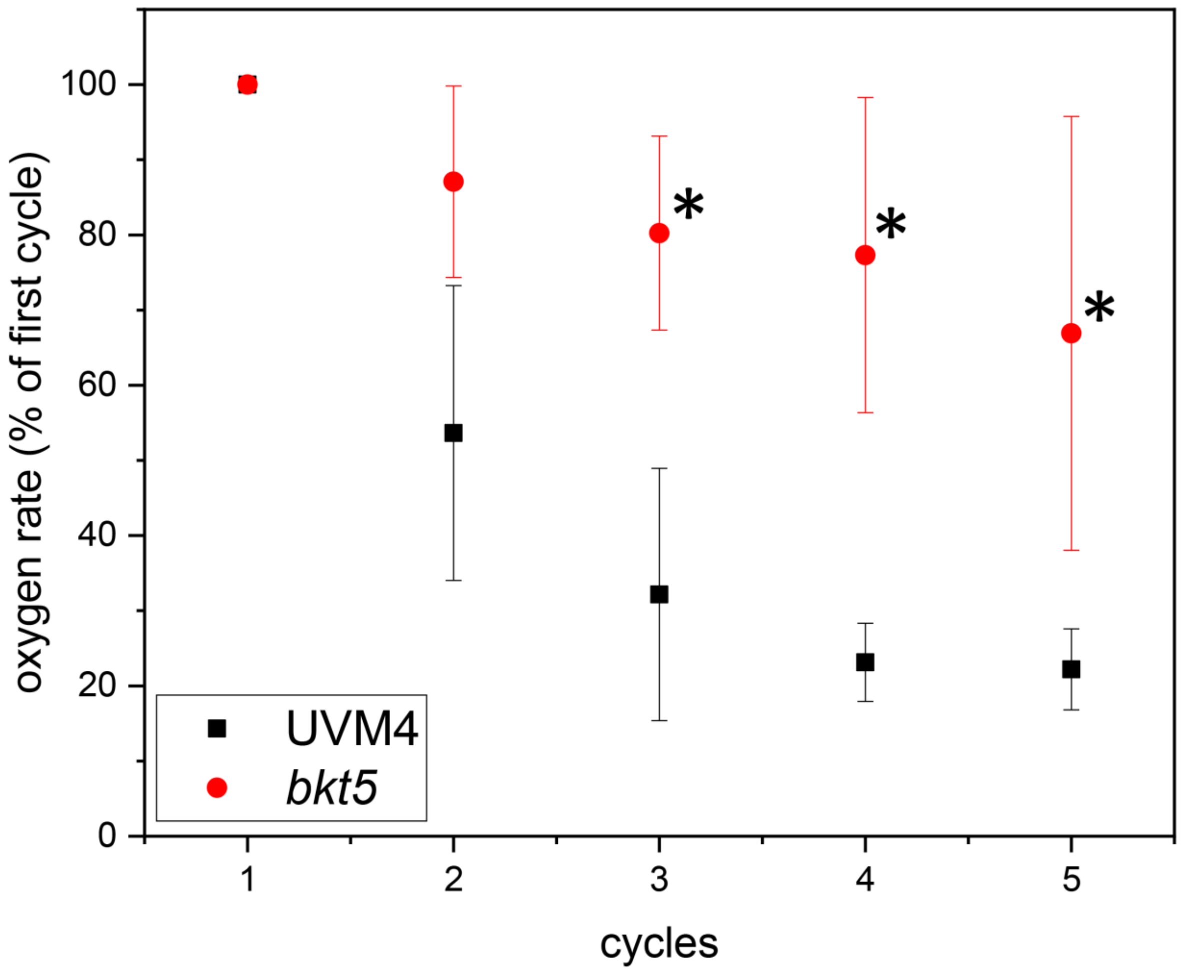

Figure S5. Rate of oxygen evolution during high light stress. Rate of oxygen evolution calculated during the different cycles of illumination as described in in Figure 4. The rate is expressed as percentage of the value in the first cycle. Error bars are reported as standard deviation (n=5). * indicates *bkt5* values that are significantly different from UVM4 (Student's test, P < 0.05).

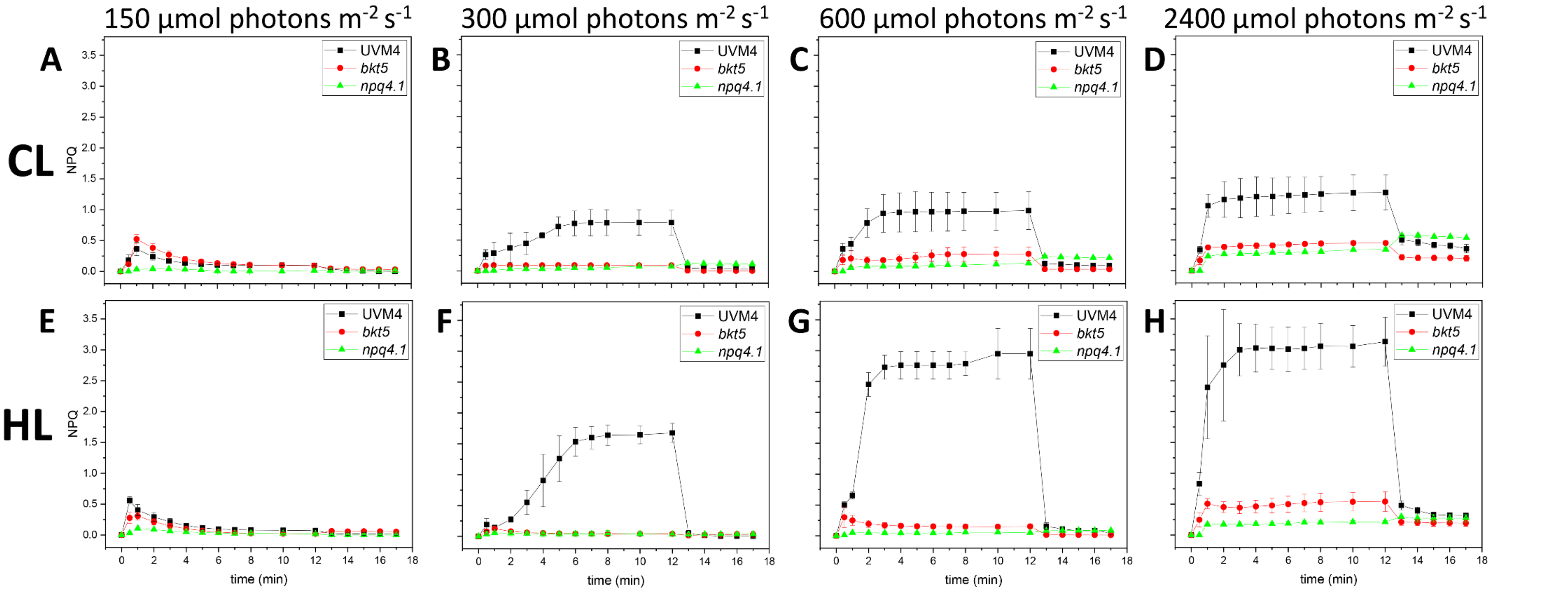

Figure S6. Nonphotochemical quenching (NPQ) at different light intensities. Cells of UVM4 (black), *bkt5* (red and npq4.1 (green), adapted in control (CL, A-D) or high light (HL, E-H) were illuminated with different actinic lights, from 150 to 2400 µmol photons m^{-2} s^{-1}, in order to obtain NPQ kinetic. Error bars are reported as standard deviation (n=4). * indicates *bkt5* values that are significantly different from UVM4 (Student's test, $P < 0.05$).

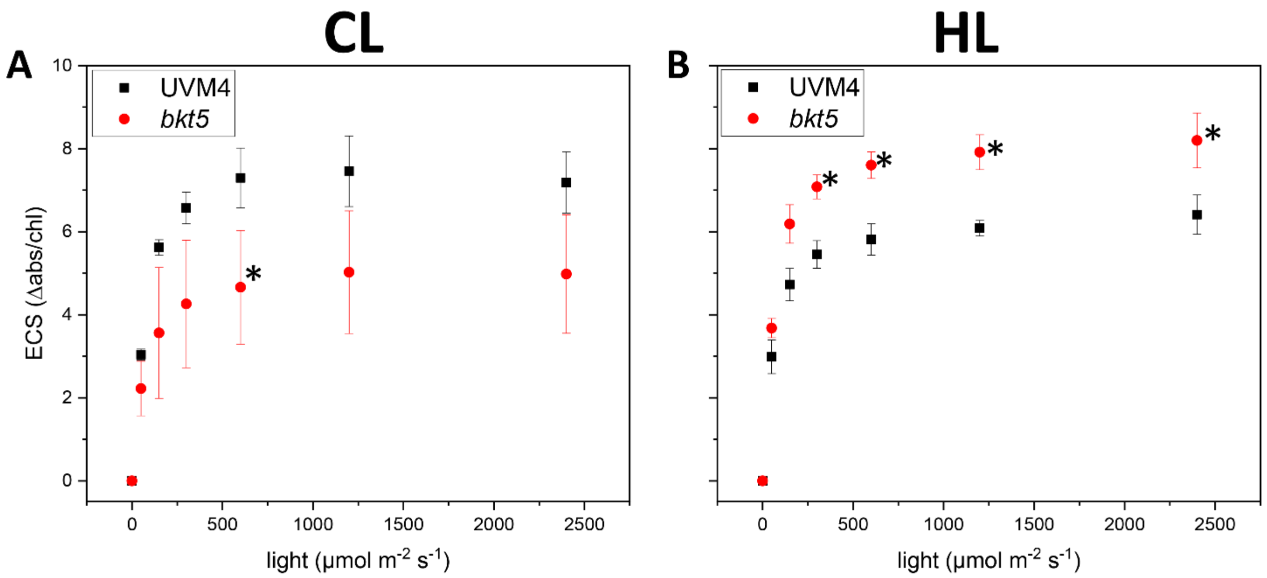

Figure S7. Estimation of total proton motive force. Proton motive force was estimated by measuring electrochromic shift of carotenoid absorption spectrum, (ECS) All measurement were performed on UVM4 (black) and *bkt5* (red) cells acclimated to control (CL, left) and high light (HL, right). Error bars are reported as standard deviation (n=4). * indicates *bkt5* values that are significantly different from UVM4 (Student's test, $P < 0.05$).

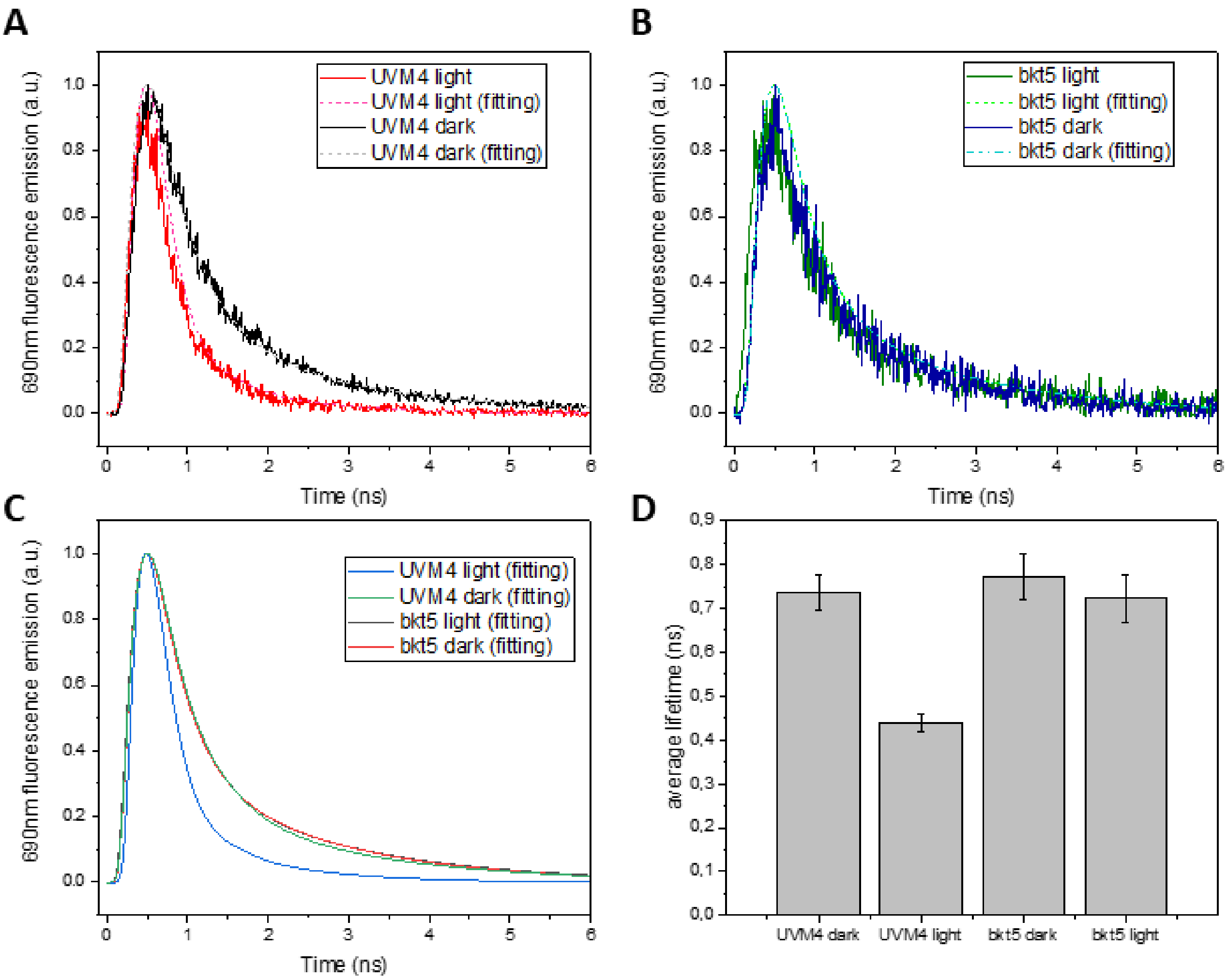

Figure S8. Time resolved fluorescence emission of whole cells at 77K. (A-B) 77K fluorescence decay kinetics lifetimes measured on HL acclimated cells of UVM4 (A) and *bkt5* (B) in dark-adapted state (dark) or after 60 min of illumination at 2400 µmol photons m^{-2} s^{-1} (light). (C) Comparison of fitting exponential curves of kinetic traces reported in Panel A-B. (D) Average fluorescence lifetimes of kinetic traces reported in Panel A-B calculated from the fitting results reported in C. Error bars are reported as standard deviation (n=4). * indicates *bkt5* values that are significantly different from UVM4 (Student's test, $P < 0.05$).

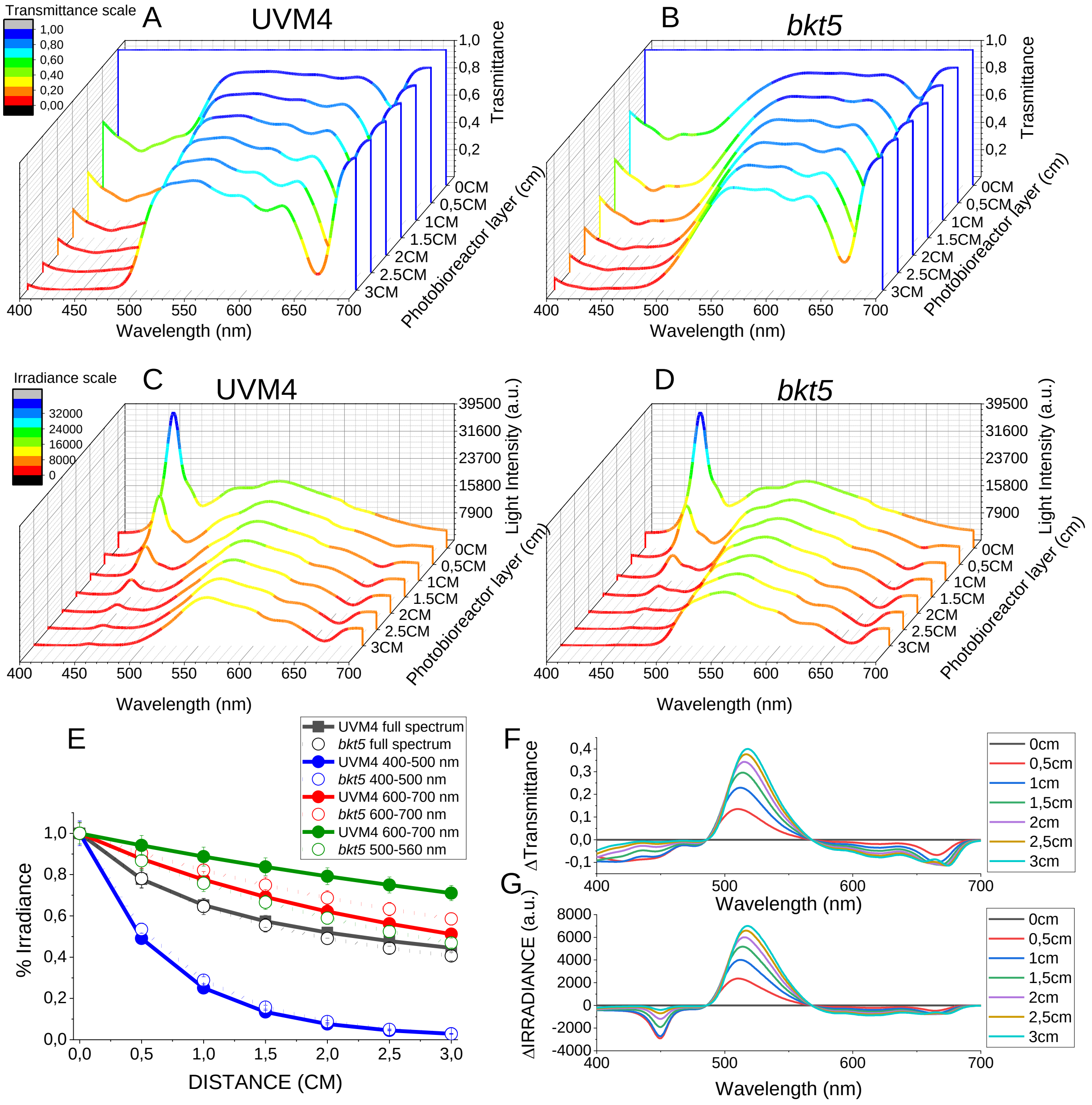

Figure S9. Transmittance and light penetration in photobioreactors. Transmittance (A. B) and light spectra (C, D) calculated for UVM4 (A, C) and *bkt5* (B, D) cell cultures at exponential phase cultivated in 3 cm wide photobioreactors used for the results reported in Figure 6 (MC-1000-OD, Photon system Instrument, Cech republic). The transmittance spectra (A, B) were calculated at different layers of photobioreactors (0, 0.5, 1, 1.5, 2, 2.5 and 3 cm) considering the absorption spectra of $1x10^5$ cells/ml and the cell density at exponential phase in photobioreactors. From the transmittance spectra the light spectra at the different layers were calculated from the spectrum of the light emitted by LEDs equipped in the photobioreactors used. The percentage of incident irradiance penetrating at the different layers for UVM4 and *bkt5* cell cultures is reported in (E) calculated as described in the following: the area of the light spectra at the different layer in the 400-700 nm (full spectrum, black lines and symbol), 400-500 nm (blue lines and symbols), 600-700 nm (red lines and symbols) and 500-560 nm (green lines and symbols) for the different layers were normalized to the 0 cm layer case (incident irradiance). (F) Difference transmittance spectra (Δ transmittance) at the different layers calculated subtracting the *bkt5* spectrum to the UVM4 spectrum. (G) Difference spectra of the light penetrating at the different layers calculated subtracting the *bkt5* spectrum to the UVM4 spectrum. Error bars are reported as standard deviation (n=4).

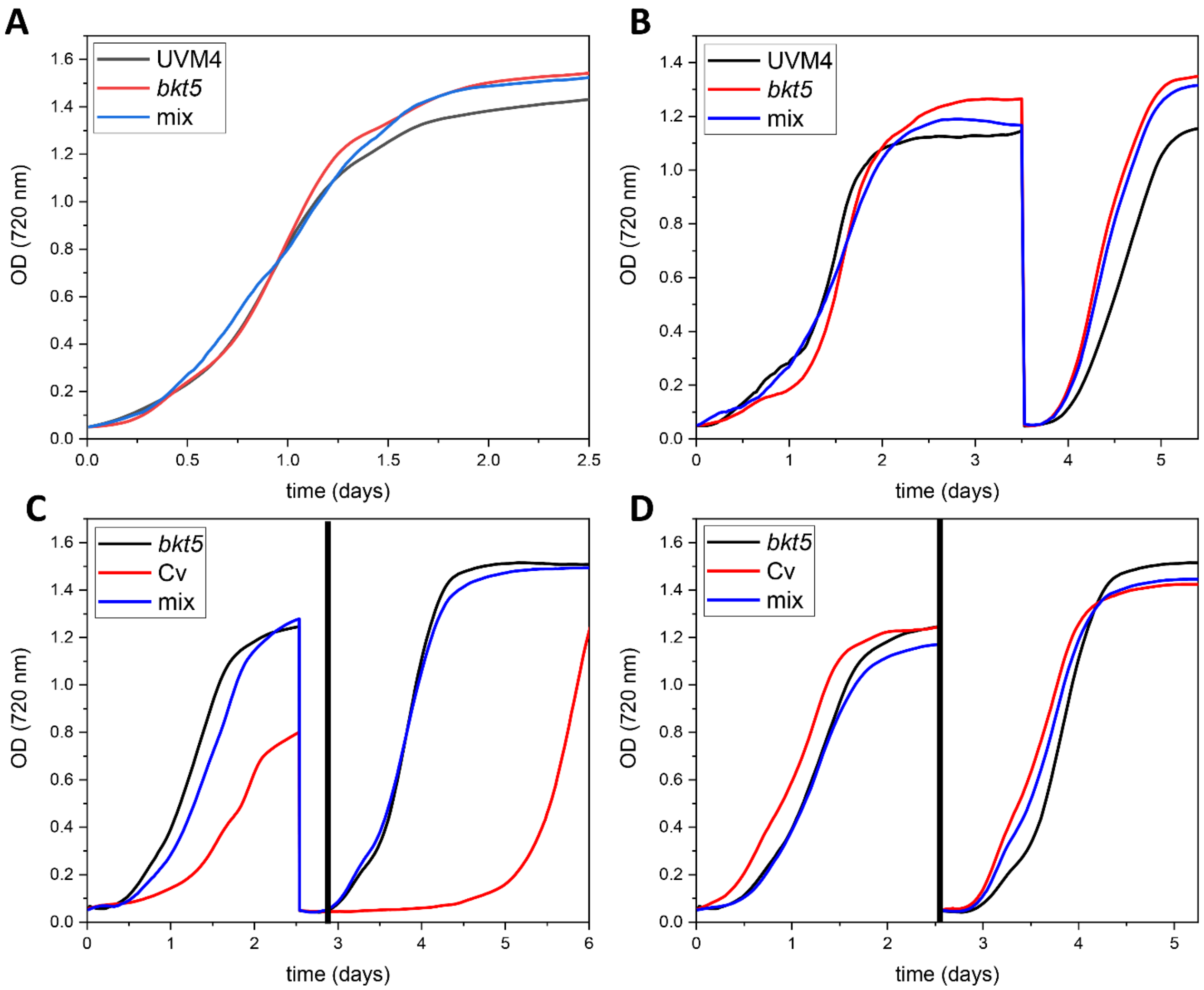

Figure S10. Growth curves of UVM4, *bkt5* and *Chlorella vulgaris* in competitive growth tests. (A,B) Growth curves starting from 1*10^6 cell/ml of UVM4 (black), *bkt5* (red) or a mix of the two genotypes in equal amount (blue), at 3000 µmol photons m^{-2} s^{-1} in TAP (A) or HS (B) as described in figure 7. (C,D) (C) Growth curves starting from 1*10^6 cell/ml of *bkt5* (black), *C. vulgaris* (Cv, red) or a mix of the two genotypes in equal cellular amount (mix, blue) at 3000 µmol photons m^{-2} s^{-1}. (D) Growth curve starting from 1*10^6 cell/ml of *bkt5* and a concentration of Cv cells that contain the same chl amount as 1*10^6 cell/ml of *bkt5* or a mix of the two genotypes in equal chlorophyll amount as described in figure 8.

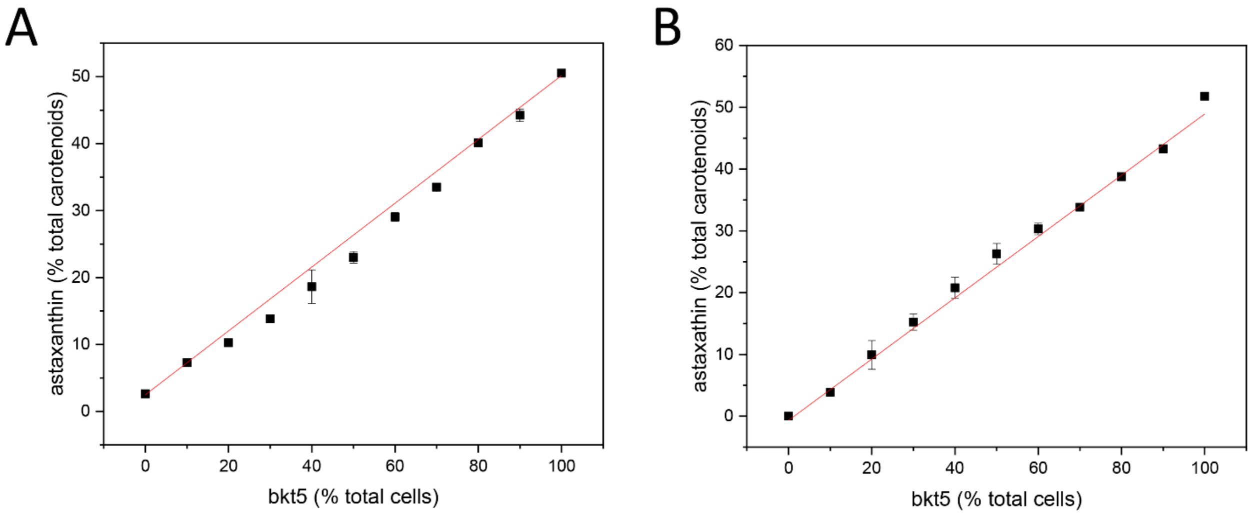

Figure S11. Titration curve of *bkt5* cells in mixture with UVM4 and *C. vulgaris* cells. (A) different percentage of *bkt5* cells, from 0 to 100%, were mixed with UVM4 cells after growth at 3000 μmol photons m^{-2} s^{-1}. Astaxanthin content, in the different combination, was quantified by fitting of the acetone extract to create the correlation (linear fitting) between astaxanthin amount and *bkt5* cells percentage. This correlation was used to estimate, from the astaxanthin content, the percentage of *bkt5* cells in mix tube of the competitive growth shown in Figure 7. (B) The same procedure was used for the competitive growth with *C. vulgaris* showed in Figure 7.

Table S1. Pigment content and Fv/Fm of UVM4 and *bkt5*. Pigments content determined in cells grown at control (CL) or high (HL) light in HS 1 week starting from $5*10^5$ cells/ml. Data are expressed as means ± SD (n = 4). * indicates *bkt5* values that are significantly different (Student's t test, P < 0.05) from UVM4. Abbreviation indicate: Chlorophyll (chl), total carotenoid (car), total ketocarotenoid (keto).

CL		pg chl/cell	Pg car/cell	chl a/b	chl/car	% keto/car	Fv/Fm
	UVM4	3.14 ± 0.34	0.98 ± 0.11	2.59 ± 0.06	3.2 ± 0.04	0 ± 0	0.76 ± 0.01
	bkt5	2.09 ± 0.21*	0.76 ± 0.08*	2.46 ± 0.06*	2.74 ± 0.06*	51.07 ± 2.90*	0.65 ± 0.02*
HL	UVM4	2.34 ± 0.03	0.89 ± 0.02	2.74 ± 0.02	2.62 ± 0.04	0 ± 0	0.72 ± 0.02
	bkt5	1.28 ± 0.06*	0.59 ± 0.03*	2.52 ± 0.05*	2.18 ± 0.05*	58.61 ± 0.80*	0.52 ± 0.04*

Table S2 Carotenoids content of UVM4 and *bkt5*. Carotenoids distribution in thylakoid membranes isolated from UVM4 and *bkt5* strain normalized to 100 chlorophylls molecules. Values are reported as moles of each pigment per 100 moles of chlorophylls. Acclimation to control light (CL) or high light (HL) was performed respectively at 100 and 600 µmol photons m^{-2} s^{-1}. Error bars are reported as standard deviation (n=4). * indicate *bkt5* values that are significantly different from UVM4 under respective light regime (Student's test, $P < 0.05$).

		neoxanthin	loroxanthin	violaxanthin	lutein	zeaxanthin	β carotene	astaxanthin	adonixanthin	adonirubin	canthaxanthin
CL	UVM4	4.76 ± 0.12	6.80 ± 0.22	4.48 ± 0.48	8.86 ± 0.82	1.30 ± 0.37	6.00 ± 1.05	-	-	-	-
	bkt5	1.00 ± 0.06*	2.64 ± 0.12*	1.58 ± 0.07*	6.36 ± 0.30*	1.14 ± 0.14	5.14 ± 0.31	13.76 ± 0.19*	2.17 ± 0.07*	1.43 ± 0.06*	1.28 ± 0.07*
HL	UVM4	2.11 ± 0.32	0.67 ± 0.03	8.51 ± 0.34	18.22 ± 0.52	5.02 ± 0.09	8.22 ± 1.3	-	-	-	-
	bkt5	1.16 ± 0.04*	0.82 ± 0.01*	1.92 ± 0.02*	11.32 ± 1.33*	2.09 ± 0.38*	4.50 ± 1.02*	18.02 ± 0.13*	2.30 ± 0.05*	3.64 ± 0.06*	8.35 ± 0.11*

Table S3. Photosynthesis and respiration rates. Parameters extrapolated from oxygen light saturation curves shown in figure 3 g-h. Data are expressed as mean ± SD (n >3). * indicates *bkt5* values that are significantly different (Student's t-test, $P < 0.05$) from UVM4.

	LL		HL	
	UVM4	*bkt5*	UVM4	*bkt5*
Respiration in the dark (O_2 pmol/cell/min)	4.31 ± 0.24	4.34 ± 1.06	4.44 ± 0.56	5.54 ± 1.01
Pmax (O_2 nmol/mg chl/h)	117.1 ± 11.4	109.4 ± 11.4	77.4 ± 27.1	139.3 ± 24.3*
Half-saturation intensity (µmol m^{-2} s^{-1})	526 ± 20	534 ± 121	354 ± 148	932 ± 178*
slope of linear increase	0.012 ± 0.001	0.011 ± 0.001	0.010 ± 0.001	0.011 ± 0.001
